# Supplementary material for: The relationship between prenatal heat exposure and birth outcomes: How much does the heat metric matter?
Source: PLoS One. 2025 Sep 3;20(9):e0330498. doi: 10.1371/journal.pone.0330498 (PMC12407402; doi:10.1371/journal.pone.0330498)
Supplement: S1 Appendix — (DOCX) [file pone.0330498.s001.docx]

**S1 Appendix: NASA satellite vs observational weather data**

The weather data we use in this paper comes from NASA satellite data, and are estimated using NASA’s MERRA-2 model.

We also have observed weather data from the Australian Bureau of Meteorology for some, but not all, parts of the NT. As a test of how well these two series relate to each other, below we present graphs plotting the NASA modelled data (blue lines) and the Bureau of Meteorology observed data (red lines) together, in Alice Springs. As is evident here, while the estimates are not exactly the same, the two series line up well. However, there are some periods without complete observational data (e.g. see figure below), in which the NASA data are preferrable. This issue is more acute in more remote areas, which is why we use the NASA data in our analysis.

| 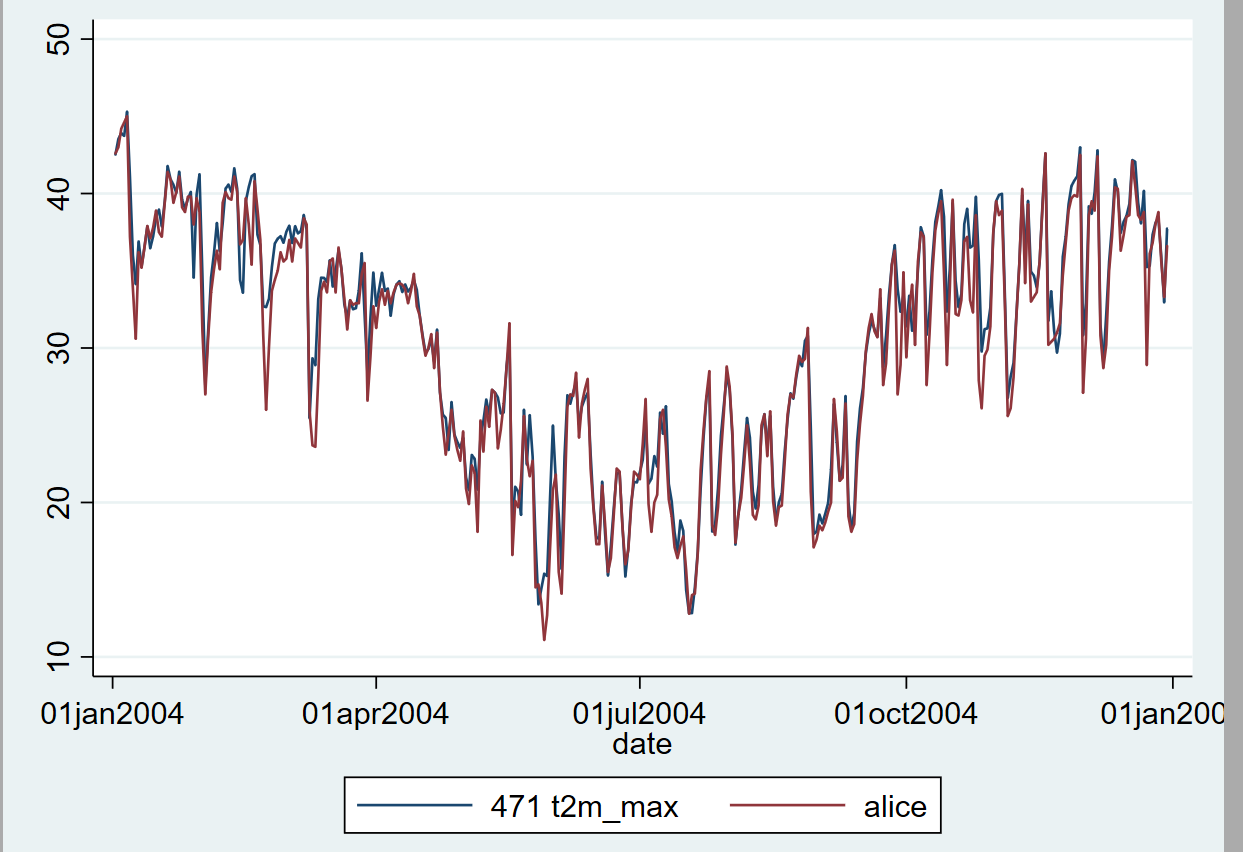   1. Maximum daily temperatures in 2004 | 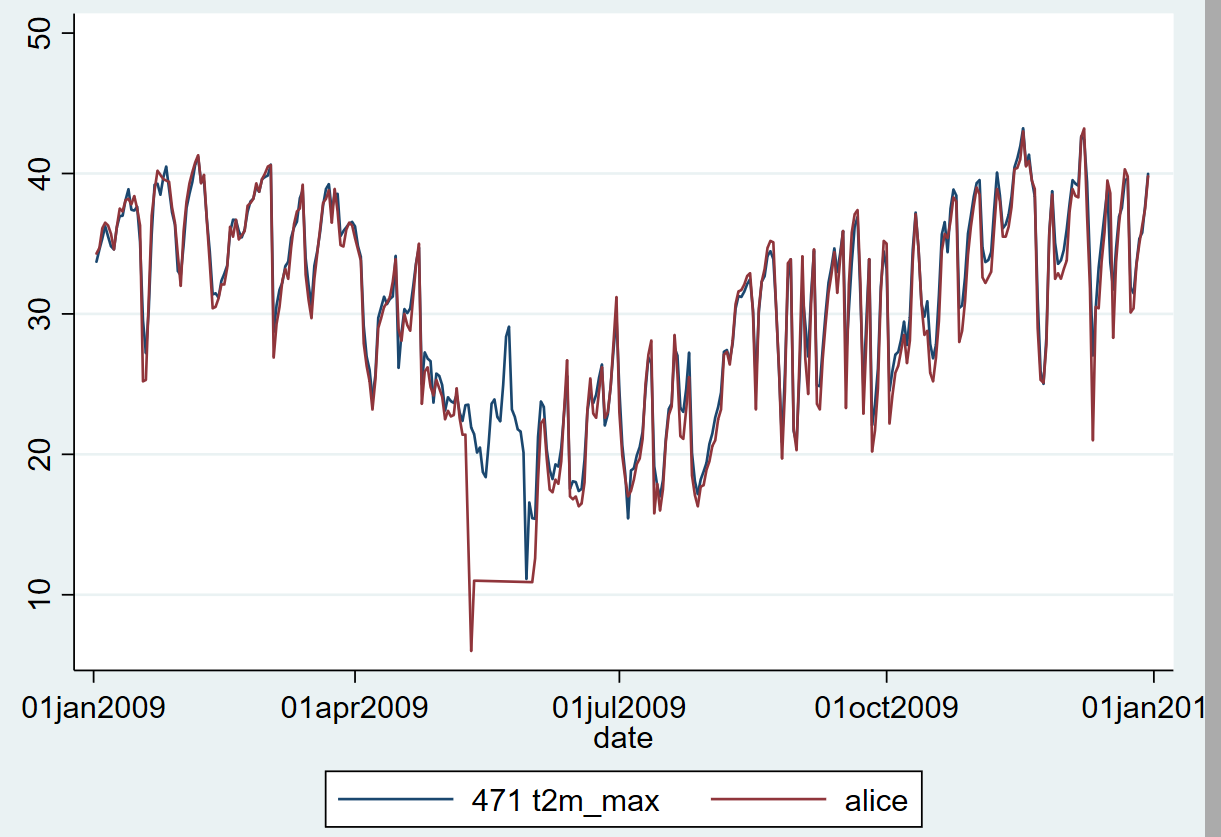   1. Maximum daily temperature in 2009 |
| --- | --- |
| 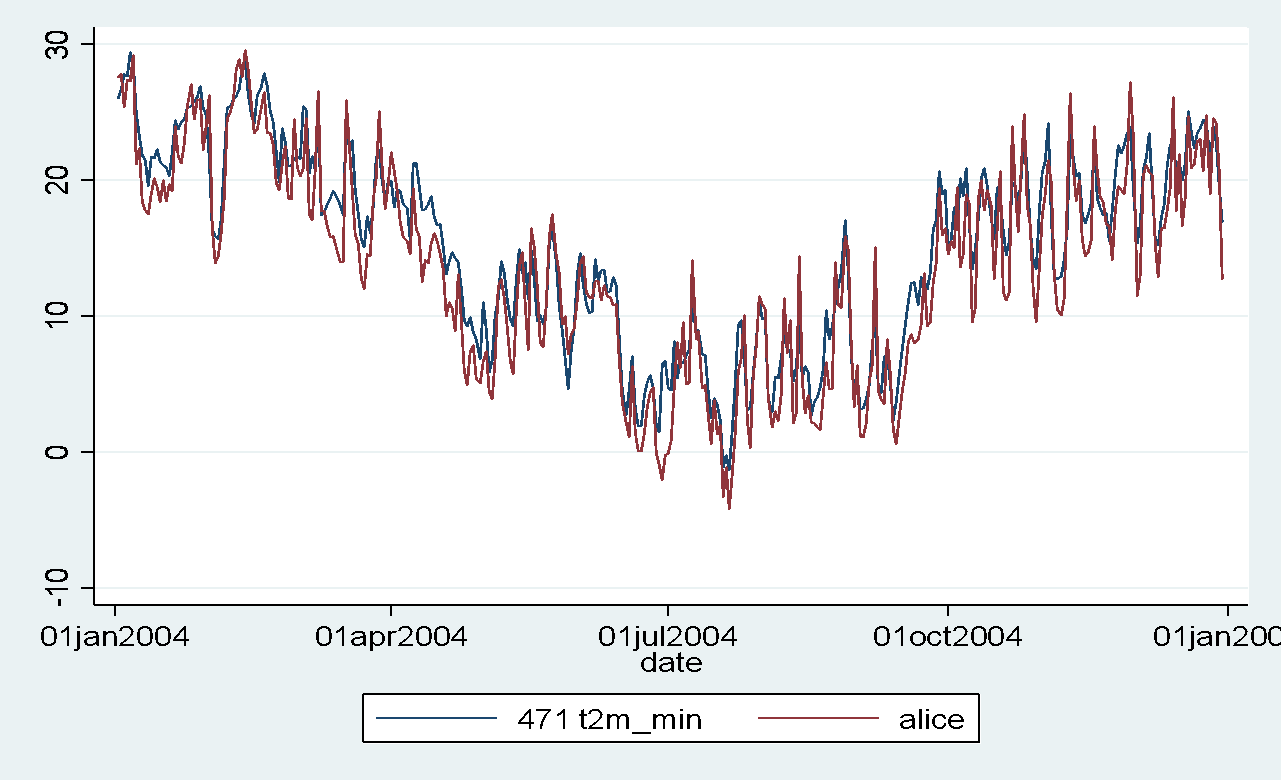   1. Minimum daily temperatures in 2004 | 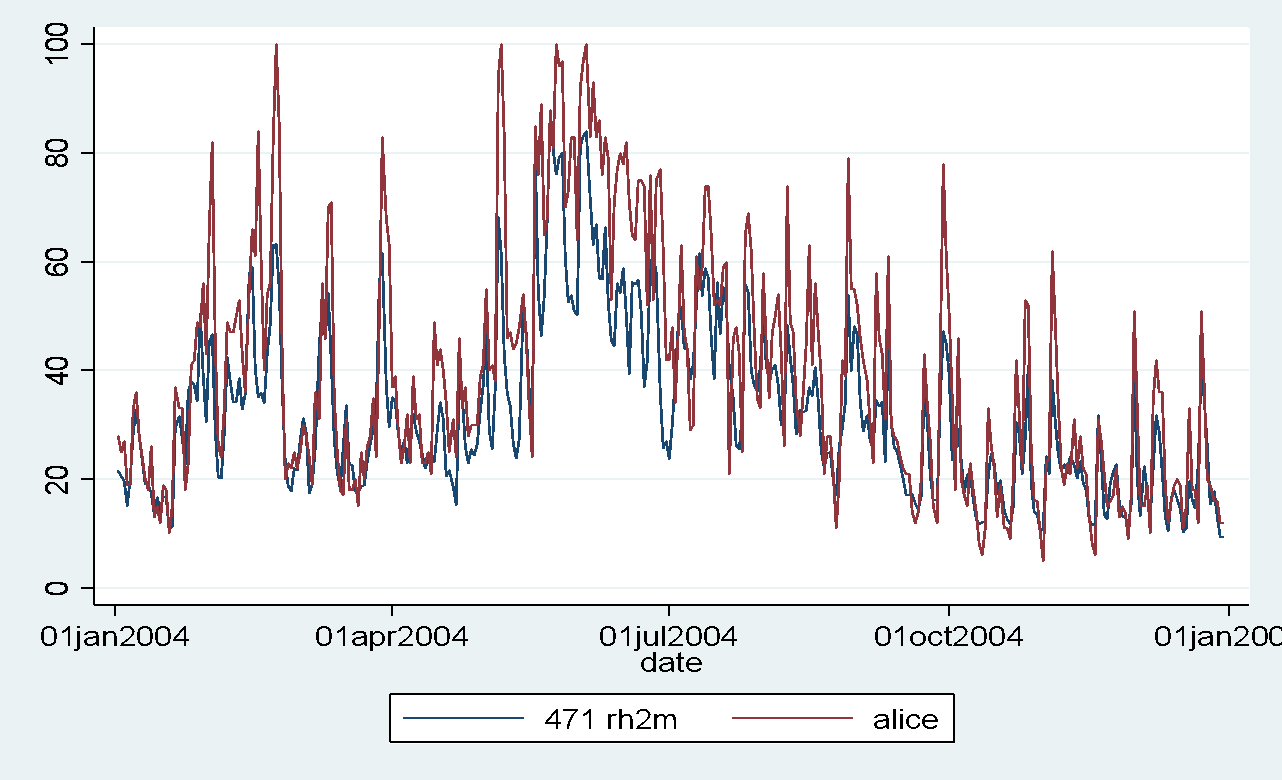   1. Relative humidity in 2004 |

**Figure: Observational and NASA data in Alice Springs**

The above figures plot NASA data (blue lines) against Australian Bureau of Meteorology data (red lines) for the same series in Alice Springs.

Source: NASA reanalysis data and Australian Bureau of Meteorology
