# Supplementary material for: The relationship between prenatal heat exposure and birth outcomes: How much does the heat metric matter?
Source: PLoS One. 2025 Sep 3;20(9):e0330498. doi: 10.1371/journal.pone.0330498 (PMC12407402; doi:10.1371/journal.pone.0330498)
Supplement: S2 Appendix — (DOCX) [file pone.0330498.s002.docx]

## S2 Appendix: Prediction methodology

We use predictions to estimate the likely rate of preterm birth if all babies were in utero during the hottest 9 months of the year, and compare this to the likely rate of preterm birth if all babies were in utero during the coolest 9 months of the year. By generating these predictions for each heat metric, we are able to compare the implications of each heat metric like-for-like.

To do this, we generate predictions for all babies in our sample, with the point estimate reported in the main text representing the average across the full sample. However, we replace the heat conditions that each baby actually experienced with heat conditions typical of the hottest (coolest) 9 months of the year in the mother’s place of residence. These typical conditions are calculated based on location-specific averages for 1996-2014 in May (for the hottest 9 months) and November (for the coolest 9 months).

These predicted values are given by:

$$\hat{preterm_{itj}}=b_{0}+\sum_{k}^{3} {b_{1}^{k}heat}_{j}^{k}+b_{2}Aboriginalstatus+b_{3}motherage+b_{4}firstpregnancy+\gamma_{my}+\theta_{jmi}$$

where we substitute the estimated regression coefficients for the ‘*b*’s and fixed effects, substitute the child’s actual characteristics for the covariates, and insert location-average values of heat exposure as ‘heat’.

As an example, consider a female baby, it is her mother’s first pregnancy, she is Aboriginal, aged between 25–29, and lives in Darwin. She was actually conceived in January 2001 and born in October 2001. Her predicted risk of preterm birth if she were instead born in May with the ‘Trimester average’ heat metric would be given by the following parameters:

| **variable** | **Coefficient** | **Value – May birth** | **Value – Nov birth** |
| --- | --- | --- | --- |
| b_0 | -0.323 |  |  |
| Heat_tri1 | 0.000585 | 32.22 | 30.04 |
| Heat_tri2 | -0.00103 | 30.66 | 29.59 |
| Heat_tri3 | 0.00691 | 30.01 | 32.11 |
| Aboriginal status | 0.0567392 | Yes | Yes |
| Mother’s age=25-29 | -0.005715 | Yes | Yes |
| First pregnancy | 0.0047182 | Yes | Yes |
| Conception month-year (FE) = Jan 2001 | 0.209 |  |  |
| Location-month-sex (FE) = Darwin, January, female | 0.002 |  |  |
| **Predicted risk of preterm birth** |  | 0.138 | 0.153 |

In this example, a baby born in November actually has a higher risk of preterm birth than a baby born in May, because this heat metric estimates that it is third trimester heat exposure which has the largest impact – and average third trimester temperatures are higher for babies born in November than those born in May.

Note also that the intercept reported here is different from the one in our main results table – this is because our main estimates are estimated using the ‘areg’ command in Stata, which does not generate estimates of each fixed effect. For the purposes of this example, we have re-estimated our model entering the fixed effects terms manually. The main coefficients are the same in both cases, but the intercept is different.

We generate these predictions for every individual in the sample, then take an average across all individuals to generate our aggregate predictions. The standard errors we report are similarly the average of all individual standard prediction errors (‘predict, stdp’ in Stata).
