# Supplementary material for: The relationship between prenatal heat exposure and birth outcomes: How much does the heat metric matter?
Source: PLoS One. 2025 Sep 3;20(9):e0330498. doi: 10.1371/journal.pone.0330498 (PMC12407402; doi:10.1371/journal.pone.0330498)
Supplement: S1 Fig — (DOCX) [file pone.0330498.s003.docx]

**Temperate climates**

| Tokyo, Japan  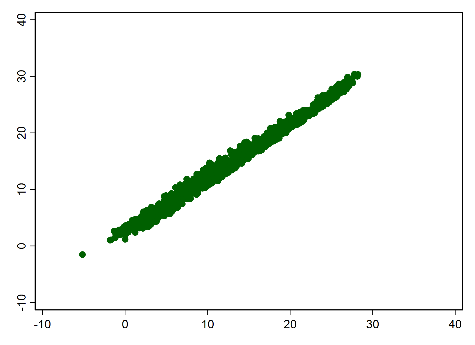 | Paris, France  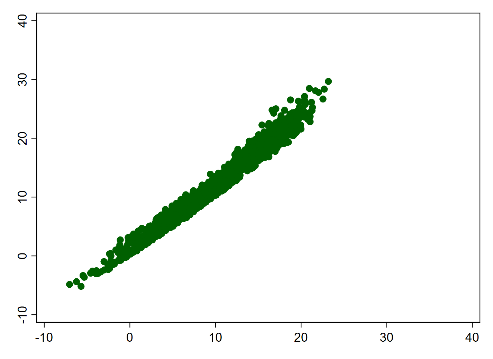 | Shanghai, China  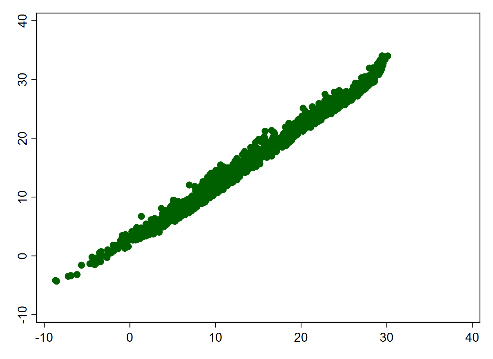 |
| --- | --- | --- |
| Istanbul, Türkiye  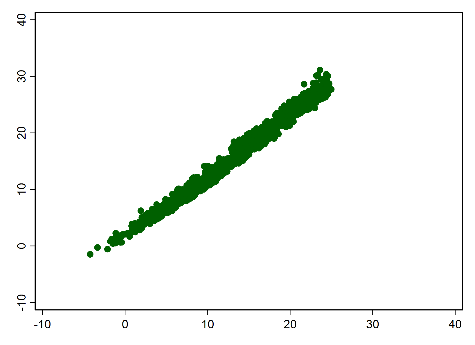 | New York, USA  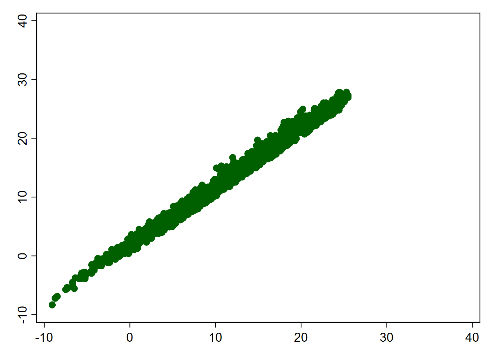 |  |
| **Tropical climates** | | |
| Darwin, Australia  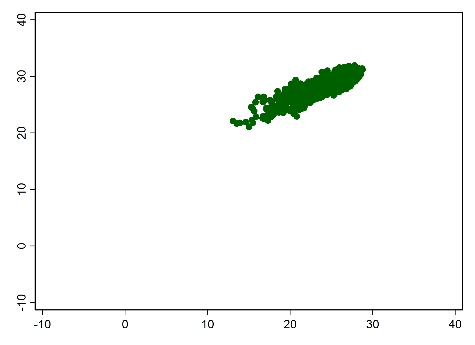 | Mexico City, Mexico  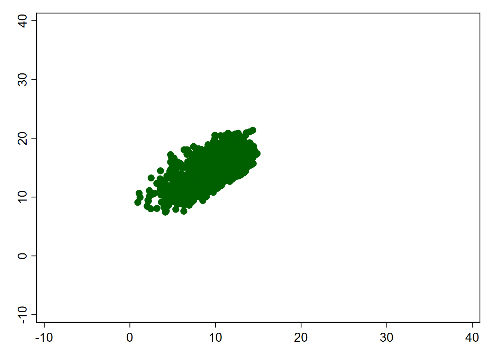 | Mumbai, India  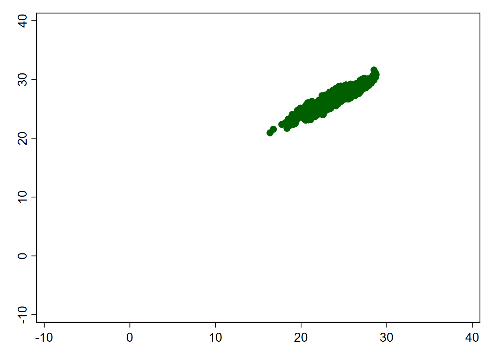 |
| Jakarta, Indonesia  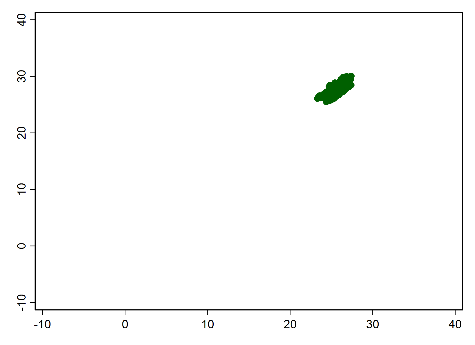 | Lagos, Nigeria  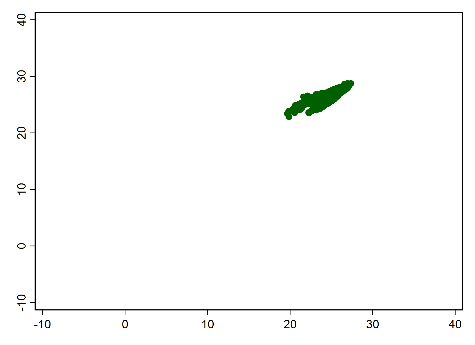 |  |

**Figure: Scatter plot of daily average air temperatures and daily average wet bulb temperatures, 2020-2023**

Source: NASA reanalysis data
