# Supplementary material for: The relationship between prenatal heat exposure and birth outcomes: How much does the heat metric matter?
Source: PLoS One. 2025 Sep 3;20(9):e0330498. doi: 10.1371/journal.pone.0330498 (PMC12407402; doi:10.1371/journal.pone.0330498)
Supplement: S2 Fig — (DOCX) [file pone.0330498.s004.docx]

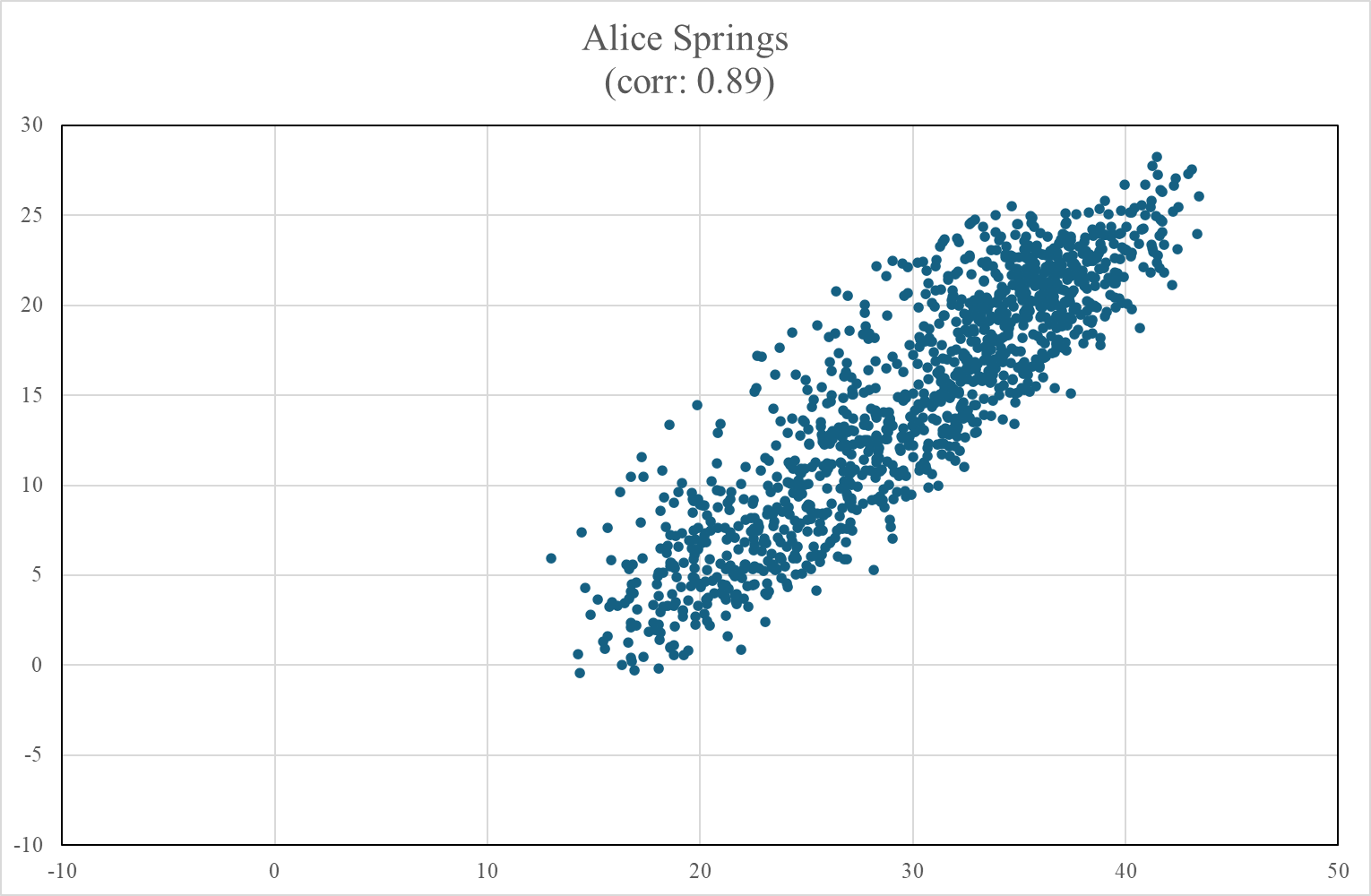


**Scatter plot of daily maximum and minimum temperature in Alice Springs, 2020-2023**

Source: NASA reanalysis data
