# Supplementary material for: The relationship between prenatal heat exposure and birth outcomes: How much does the heat metric matter?
Source: PLoS One. 2025 Sep 3;20(9):e0330498. doi: 10.1371/journal.pone.0330498 (PMC12407402; doi:10.1371/journal.pone.0330498)
Supplement: S3 Fig — (DOCX) [file pone.0330498.s005.docx]

**Scatterplot of daily rainfall with average wet bulb temperature and maximum temperature in Darwin**

| 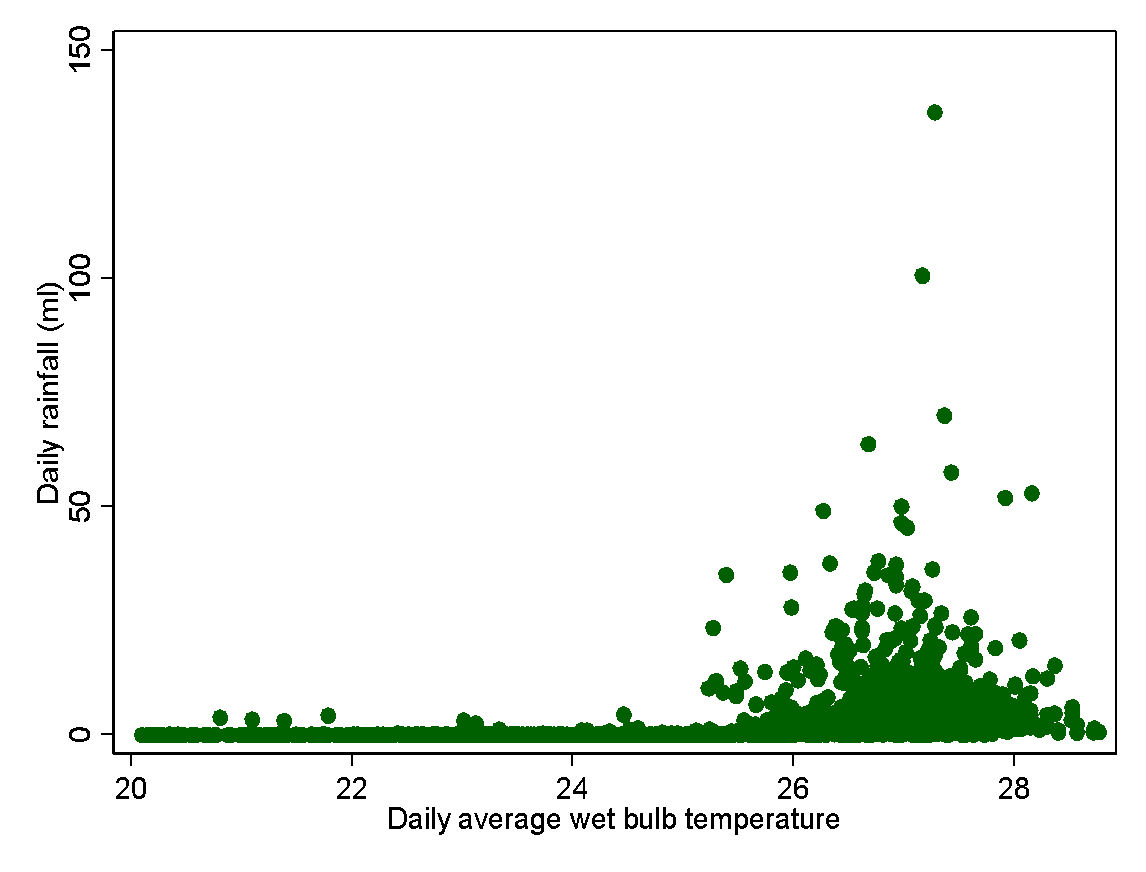 | 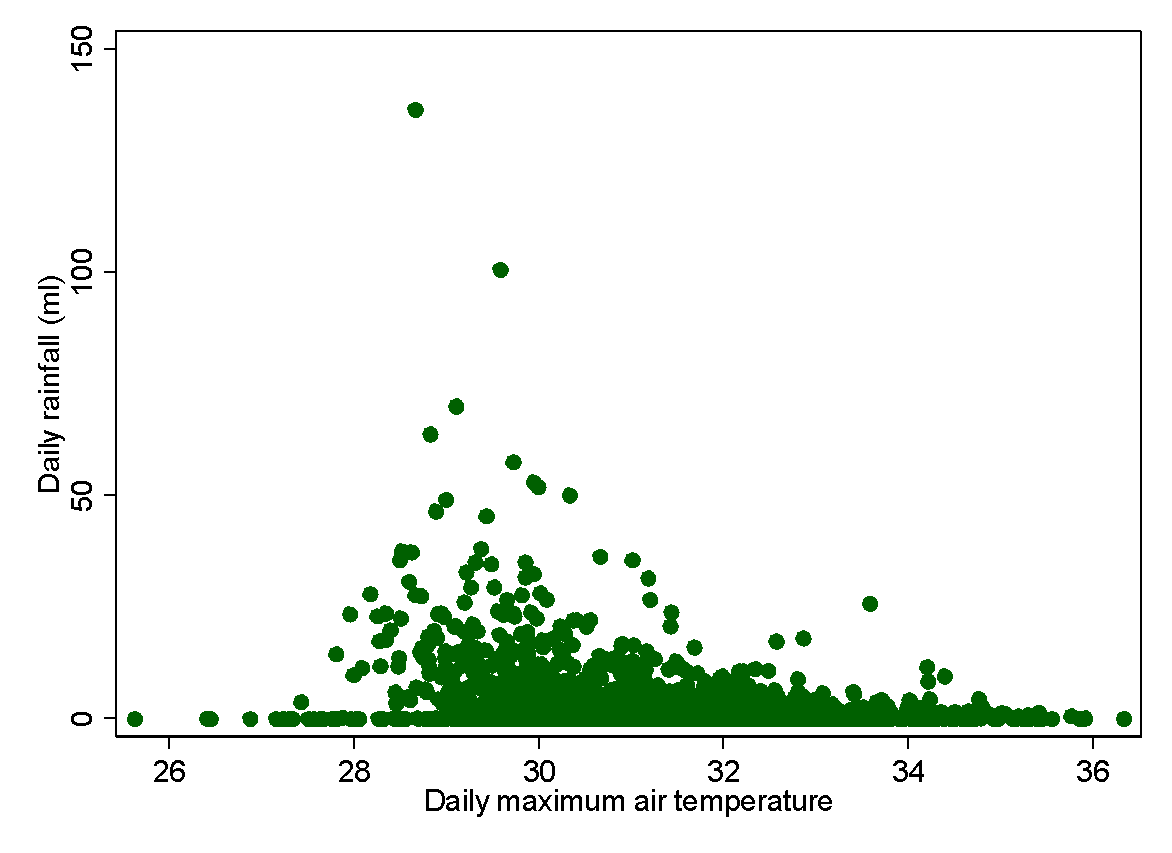 |
| --- | --- |

Source: NASA January 2020 to December 2023
