# Supplementary material for: The relationship between prenatal heat exposure and birth outcomes: How much does the heat metric matter?
Source: PLoS One. 2025 Sep 3;20(9):e0330498. doi: 10.1371/journal.pone.0330498 (PMC12407402; doi:10.1371/journal.pone.0330498)
Supplement: S1 Table — (DOCX) [file pone.0330498.s006.docx]

**S1 table: Regression estimates with alternative specifications of fixed effects**

|  |  | (1) | (2) | (3) | (4) | (5) |
| --- | --- | --- | --- | --- | --- | --- |
| **1st trimester** | <20 | 0.000 | 0.000 | 0.000 | -0.000 | -0.000 |
|  |  | (0.001) | (0.001) | (0) | (0) | (0) |
|  | 20-25 | 0.001 | 0.001 | -0.000 | 0.000 | 0.000 |
|  |  | (0) | (0.001) | (0.001) | (0.001) | (0.001) |
|  | 30-35 | 0.001** | 0.001*** | 0.000** | 0.000 | 0.000*** |
|  |  | (0.000) | (0.000) | (0.000) | (0.000) | (0.000) |
|  | 35-40 | 0.001** | 0.001** | 0.001** | 0.000 | 0.001** |
|  |  | (0.000) | (0.000) | (0.000) | (0.000) | (0.000) |
|  | 40+ | 0.000 | 0.000 | 0.001* | 0.000 | 0.000 |
|  |  | (0.001) | (0.001) | (0.000) | (0.000) | (0.000) |
|  |  |  |  |  |  |  |
| **2nd trimester** | <20 | -0.001* | -0.002** | -0.001 | 0.000 | -0.001 |
|  |  | (0.001) | (0.001) | (0.001) | (0.001) | (0.000) |
|  | 20-25 | -0.001*** | -0.001*** | 0.000 | 0.000 | 0.000 |
|  |  | (0.000) | (0.000) | (0.000) | (0.000) | (0.000) |
|  | 30-35 | 0.000 | 0.000 | 0.000 | 0.000 | 0.000 |
|  |  | (0.000) | (0.000) | (0.000) | (0.000) | (0.000) |
|  | 35-40 | -0.000 | -0.000 | -0.000 | -0.000 | -0.000 |
|  |  | (0.000) | (0.000) | (0.000) | (0.000) | (0.000) |
|  | 40+ | -0.000 | -0.000 | 0.000 | 0.000 | 0.000 |
|  |  | (0.000) | (0.000) | (0.000) | (0.000) | (0.000) |
|  |  |  |  |  |  |  |
| **3rd trimester** | <20 | -0.003*** | -0.003*** | -0.000 | -0.000 | -0.000 |
|  |  | (0.001) | (0.001) | (0) | (0) | (0) |
|  | 20-25 | -0.002** | -0.001 | -0.001* | -0.001 | -0.001 |
|  |  | (0.001) | (0.001) | (0.001) | (0.001) | (0.001) |
|  | 30-35 | 0.000* | 0.000* | -0.000 | -0.000 | -0.000 |
|  |  | (0.000) | (0.000) | (0.000) | (0.000) | (0.000) |
|  | 35-40 | 0.000 | 0.000 | 0.000 | 0.000 | 0.000 |
|  |  | (0.000) | (0.000) | (0.000) | (0.000) | (0.000) |
|  | 40+ | 0.001 | 0.001 | 0.001 | 0.001 | 0.001 |
|  |  | (0.001) | (0.001) | (0.001) | (0.001) | (0.001) |
|  |  |  |  |  |  |  |
| N |  | 34258 | 34258 | 34258 | 34258 | 34258 |
| R-sq |  | 0.086 | 0.058 | 0.026 | 0.023 | 0.023 |
| **Fixed effects** |  |  |  |  |  |  |
| Month |  |  |  |  | x | x |
| Year |  |  |  |  | x |  |
| Month-year |  | x | x | x |  |  |
| Cluster |  |  |  | x | x | x |
| Cluster-month |  |  | x |  |  |  |
| Cluster-month-sex |  | x |  |  |  |  |

This table shows regression estimates using the model specified in equation (1), with our benchmark heat metric. Each column presents estimates from a different approach to specifying the time and location fixed effects. Cluster-robust standard errors in parentheses.
