# Supplementary material for: The relationship between prenatal heat exposure and birth outcomes: How much does the heat metric matter?
Source: PLoS One. 2025 Sep 3;20(9):e0330498. doi: 10.1371/journal.pone.0330498 (PMC12407402; doi:10.1371/journal.pone.0330498)
Supplement: S2 Table — (DOCX) [file pone.0330498.s007.docx]

**S2 table: Model build with multiple metrics together**

|  | (1) | (2) | (3) | (4) |
| --- | --- | --- | --- | --- |
| Statistical significance: F-test of joint significance p-value) | | | | |
| Max temp | 0.000 | 0.000 | 0.000 | 0.000 |
| Min temp |  | 0.000 | 0.009 | 0.013 |
| Wet bulb max |  |  | 0.003 | 0.010 |
| Heatwaves |  |  |  | 0.736 |
|  |  |  |  |  |
| Model fit |  |  |  |  |
| RMSE | 0.301 | 0.300 | 0.300 | 0.300 |
| Adjusted R-squared | 0.036 | 0.037 | 0.037 | 0.037 |

This table shows p-values from an F-test of joint significance of the regression coefficients of the heat metric shown. Each column represents a single regression, which additional metrics sequentially added to the model. Column 1 represents the benchmark metric and column 2 represents the preferred, ‘max and min’ metric. When added to the max and min metric, a maximum wet bulb metric have statistically significant coefficients but does not enhance model fit. The addition of the heatwave metric produces coefficients that are not statistically significant and have no impact on model fit.
