# Supplementary material for: The relationship between prenatal heat exposure and birth outcomes: How much does the heat metric matter?
Source: PLoS One. 2025 Sep 3;20(9):e0330498. doi: 10.1371/journal.pone.0330498 (PMC12407402; doi:10.1371/journal.pone.0330498)
Supplement: S3 Table — (DOCX) [file pone.0330498.s008.docx]

**S3 Table: regression coefficients for benchmark metric and additional outcomes**

|  |  | Preterm birth | Birthweight (grams) | Small for Gestational Age | Apgar 5 score | Admitted to Special Care Nursery |
| --- | --- | --- | --- | --- | --- | --- |
| 1^st^ trimester | Max <20 | -0.000 | 0.493 | -0.001 | -0.003 | 0.002*** |
|  |  | (0.001) | (1.217) | (0.000) | (0.002) | (0.001) |
|  | Max 20-25 | 0.001 | -0.507 | -0.000 | 0.001 | -0.000 |
|  |  | (0.000) | (1.098) | (0.001) | (0.002) | (0.001) |
|  | Max 30-35 | 0.001** | -0.813*** | 0.000 | 0.000 | 0.001*** |
|  |  | (0.000) | (0.212) | (0.000) | (0.001) | (0.000) |
|  | Max 35-40 | 0.001** | -0.341 | 0.001** | -0.000 | 0.001*** |
|  |  | (0.000) | (0.269) | (0.000) | (0.001) | (0.000) |
|  | Max 40+ | 0.000 | -1.160 | 0.002*** | 0.003 | -0.000 |
|  |  | (0.001) | (0.855) | (0.000) | (0.004) | (0.001) |
|  |  |  |  |  |  |  |
| 2^nd^ trimester | Max <20 | -0.001* | 2.911* | -0.001 | 0.000 | -0.001 |
|  |  | (0.001) | (1.518) | (0.001) | (0.003) | (0.001) |
|  | Max 20-25 | -0.001*** | 0.781 | 0.000 | 0.000 | -0.001 |
|  |  | (0.000) | (0.975) | (0.001) | (0.002) | (0.001) |
|  | Max 30-35 | 0.000 | -0.535** | 0.000* | 0.000 | 0.000 |
|  |  | (0.000) | (0.229) | (0.000) | (0.001) | (0.000) |
|  | Max 35-40 | -0.000 | 0.144 | 0.000 | -0.000 | 0.000 |
|  |  | (0.000) | (0.207) | (0.000) | (0.001) | (0.000) |
|  | Max 40+ | -0.000 | 0.159 | -0.001* | 0.002 | 0.000 |
|  |  | (0.000) | (0.695) | (0.000) | (0.002) | (0.001) |
|  |  |  |  |  |  |  |
| 3^rd^ trimester | Max <20 | -0.003*** | 3.477*** | -0.000 | 0.008** | 0.001 |
|  |  | (0.001) | (0.906) | (0.001) | (0.003) | (0.001) |
|  | Max 20-25 | -0.002** | 1.176 | 0.000 | -0.001 | -0.002* |
|  |  | (0.001) | (1.151) | (0.001) | (0.003) | (0.001) |
|  | Max 30-35 | 0.000* | -0.600** | -0.000 | -0.001 | 0.000*** |
|  |  | (0.000) | (0.247) | (0.000) | (0.001) | (0.000) |
|  | Max 35-40 | 0.000 | -0.669 | -0.000 | -0.001 | 0.000** |
|  |  | (0.000) | (0.452) | (0.000) | (0.001) | (0.000) |
|  | Max 40+ | 0.001 | -1.079 | -0.000 | -0.005 | 0.001 |
|  |  | (0.001) | (1.178) | (0.001) | (0.004) | (0.001) |
|  |  |  |  |  |  |  |
|  |  |  |  |  |  |  |
| Constant | | 0.040** | 3,404.696*** | 0.079*** | 8.972*** | 0.036* |
|  |  | (0.018) | (40.737) | (0.018) | (0.092) | (0.021) |
|  |  |  |  |  |  |  |
| N |  | 34,258 | 34,258 | 34,258 | 34,258 | 34,258 |
| R-sq |  | 0.086 | 0.125 | 0.087 | 0.080 | 0.088 |
| This table shows the regression coefficients and cluster-robust standard errors in parentheses from the model specified in equation (1) using the benchmark heat metric. Estimates are shown for preterm birth and four other measures of health at birth. As specified in equation (1), the regressions also include covariates (mother’s age, Aboriginal status, whether mother’s first pregnancy), month-year fixed effects and location-month-sex fixed effects (these are absorbed using the Stata ‘areg’, which affects the intercept but not the coefficients). | | | | | | |
