# Supplementary material for: The relationship between prenatal heat exposure and birth outcomes: How much does the heat metric matter?
Source: PLoS One. 2025 Sep 3;20(9):e0330498. doi: 10.1371/journal.pone.0330498 (PMC12407402; doi:10.1371/journal.pone.0330498)
Supplement: S7 Table — (DOCX) [file pone.0330498.s012.docx]

**S7 Table: Regression estimates for probability of preterm birth by Aboriginal status, preferred metric**

|  |  | Aboriginal | Non-Aboriginal |
| --- | --- | --- | --- |
| 1st tri | Max <20 | 0.002 | 0.000 |
|  |  | (0.001) | (0.001) |
|  | Max 20-25 | 0.002** | 0.002*** |
|  |  | (0.001) | (0.001) |
|  | Max 30-35 | 0.000 | 0.000 |
|  |  | (0.000) | (0.000) |
|  | Max 35-40 | 0.000 | 0.001*** |
|  |  | (0.001) | (0.000) |
|  | Max 40+ | -0.000 | -0.002** |
|  |  | (0.001) | (0.001) |
|  | Min <5 | -0.001 | -0.002** |
|  |  | (0.002) | (0.001) |
|  | Min 5-10 | -0.002 | -0.001* |
|  |  | (0.002) | (0.001) |
|  | Min 10-15 | -0.000 | 0.000 |
|  |  | (0.001) | (0.001) |
|  | Min 20-25 | 0.001 | 0.001*** |
|  |  | (0.001) | (0.000) |
|  | Min 25+ | 0.001 | 0.001*** |
|  |  | (0.001) | (0.000) |
| 2nd tri | Max <20 | -0.003 | -0.002 |
|  |  | (0.002) | (0.001) |
|  | Max 20-25 | -0.002 | -0.002** |
|  |  | (0.001) | (0.001) |
|  | Max 30-35 | -0.000 | 0.000** |
|  |  | (0.000) | (0.000) |
|  | Max 35-40 | 0.000 | 0.001*** |
|  |  | (0.001) | (0.000) |
|  | Max 40+ | -0.001 | 0.000 |
|  |  | (0.001) | (0.000) |
|  | Min <5 | 0.000 | 0.002 |
|  |  | (0.002) | (0.001) |
|  | Min 5-10 | 0.000 | 0.002** |
|  |  | (0.002) | (0.001) |
|  | Min 10-15 | 0.001 | 0.001* |
|  |  | (0.001) | (0.001) |
|  | Min 20-25 | 0.000 | 0.000 |
|  |  | (0.001) | (0.001) |
|  | Min 25+ | 0.001 | 0.001 |
|  |  | (0.001) | (0.001) |
| 3rd tri | Max <20 | -0.007*** | -0.000 |
|  |  | (0.003) | (0.001) |
|  | Max 20-25 | -0.004** | 0.002 |
|  |  | (0.002) | (0.002) |
|  | Max 30-35 | 0.000 | 0.000** |
|  |  | (0.000) | (0.000) |
|  | Max 35-40 | 0.002*** | 0.000 |
|  |  | (0.001) | (0.001) |
|  | Max 40+ | 0.001 | 0.002 |
|  |  | (0.001) | (0.001) |
|  | Min <5 | 0.003 | -0.002*** |
|  |  | (0.002) | (0.000) |
|  | Min 5-10 | 0.002 | -0.001 |
|  |  | (0.002) | (0.001) |
|  | Min 10-15 | 0.001 | 0.002*** |
|  |  | (0.001) | (0.001) |
|  | Min 20-25 | -0.000 | 0.000 |
|  |  | (0.001) | (0.000) |
|  | Min 25+ | -0.000 | 0.000** |
|  |  | (0.001) | (0.000) |
|  | Constant | 0.024 | -0.153*** |
|  |  | (0.063) | (0.047) |
|  | N |  |  |
|  |  | 14,646 | 19,612 |
|  | R-sq | 0.132 | 0.042 |

Note: this table shows the regression coefficients and cluster-robust standard errors in parentheses from the model specified in equation (1) using the Max + Min heat metric, with the sample split by whether the child is Aboriginal or not. The outcome is preterm birth. As specified in equation (1), the regressions also include covariates (mother’s age, Aboriginal status, whether mother’s first pregnancy), month-year fixed effects and location-month-sex fixed effects (these are absorbed using the Stata ‘areg’, which affects the intercept but not the coefficients).
