# Supplementary material for: The relationship between prenatal heat exposure and birth outcomes: How much does the heat metric matter?
Source: PLoS One. 2025 Sep 3;20(9):e0330498. doi: 10.1371/journal.pone.0330498 (PMC12407402; doi:10.1371/journal.pone.0330498)
Supplement: S8 Table — (DOCX) [file pone.0330498.s013.docx]

**S8 Table: regression coefficients for wet bulb metric with additional outcomes**

|  |  | Preterm birth | Birthweight (grams) | Small for Gestational Age | Apgar 5 score | Special Care Nursery |
| --- | --- | --- | --- | --- | --- | --- |
| 1st trimester | WB max <10 | 0.001 | 6.253 | -0.000 | 0.011 | -0.000 |
|  |  | (0.002) | (4.215) | (0.003) | (0.008) | (0.002) |
|  | WB max 10-15 | -0.001 | 3.073 | 0.003 | 0.007 | -0.001 |
|  |  | (0.002) | (2.472) | (0.002) | (0.006) | (0.002) |
|  | WB max 15-20 | -0.000 | 1.750 | 0.002 | 0.008 | -0.001 |
|  |  | (0.001) | (1.532) | (0.001) | (0.005) | (0.001) |
|  | WB max 25-30 | -0.000 | 0.218 | 0.000 | 0.000 | -0.000 |
|  |  | (0.000) | (0.422) | (0.000) | (0.001) | (0.000) |
|  | WB max 30+ | 0.000 | 0.289 | 0.000 | 0.001 | 0.000 |
|  |  | (0.001) | (1.126) | (0.001) | (0.003) | (0.001) |
|  | WB avg <10 | 0.000 | -1.802 | -0.003* | -0.005 | -0.000 |
|  |  | (0.001) | (2.249) | (0.001) | (0.005) | (0.001) |
|  | WB avg 10-15 | 0.000 | -1.497 | -0.001 | -0.002 | -0.000 |
|  |  | (0.001) | (1.045) | (0.001) | (0.005) | (0.001) |
|  | WB avg 20-25 | 0.000 | -0.904 | -0.000 | 0.000 | 0.000 |
|  |  | (0.001) | (0.621) | (0.000) | (0.001) | (0.001) |
|  | WB avg 25+ | 0.000 | -0.237 | -0.001 | 0.002 | 0.001 |
|  |  | (0.001) | (0.654) | (0.000) | (0.002) | (0.001) |
| 2nd trimester | WB max <10 | 0.000 | -1.603 | -0.001 | -0.015** | -0.004* |
|  |  | (0.002) | (3.655) | (0.002) | (0.007) | (0.002) |
|  | WB max 10-15 | -0.000 | 1.829 | -0.002 | 0.002 | -0.003 |
|  |  | (0.001) | (2.574) | (0.002) | (0.007) | (0.002) |
|  | WB max 15-20 | 0.000 | -0.687 | -0.001 | -0.005 | -0.001 |
|  |  | (0.001) | (1.826) | (0.001) | (0.004) | (0.001) |
|  | WB max 25-30 | 0.000 | -0.690 | 0.000 | -0.001 | 0.000 |
|  |  | (0.000) | (0.699) | (0.000) | (0.001) | (0.000) |
|  | WB max 30+ | 0.000 | -0.217 | 0.001** | 0.001 | 0.002*** |
|  |  | (0.001) | (0.884) | (0.000) | (0.003) | (0.001) |
|  | WB avg <10 | -0.000 | -0.151 | 0.001 | 0.002 | 0.002 |
|  |  | (0.001) | (2.392) | (0.002) | (0.006) | (0.002) |
|  | WB avg 10-15 | -0.002* | 0.894 | -0.000 | 0.005 | -0.000 |
|  |  | (0.001) | (1.623) | (0.001) | (0.003) | (0.001) |
|  | WB avg 20-25 | -0.001* | -0.362 | -0.000 | 0.002 | -0.001 |
|  |  | (0.000) | (1.071) | (0.000) | (0.001) | (0.001) |
|  | WB avg 25+ | -0.001 | -0.164 | -0.000 | 0.004** | -0.001** |
|  |  | (0.001) | (1.100) | (0.000) | (0.002) | (0.000) |
| 3rd trimester | WB max <10 | 0.000 | -1.572 | 0.000 | -0.000 | 0.001 |
|  |  | (0.002) | (3.346) | (0.002) | (0.008) | (0.002) |
|  | WB max 10-15 | -0.003 | 2.795 | 0.002* | 0.011* | -0.001 |
|  |  | (0.002) | (3.254) | (0.001) | (0.006) | (0.003) |
|  | WB max 15-20 | 0.001 | 0.001 | 0.000 | -0.003 | -0.001 |
|  |  | (0.001) | (2.447) | (0.001) | (0.005) | (0.001) |
|  | WB max 25-30 | -0.001** | 0.455 | 0.000 | 0.003** | -0.001** |
|  |  | (0.000) | (0.406) | (0.000) | (0.001) | (0.000) |
|  | WB max 30+ | 0.000 | -0.870 | -0.000 | 0.002 | 0.000 |
|  |  | (0.001) | (2.142) | (0.001) | (0.002) | (0.001) |
|  | WB avg <10 | 0.001 | -0.276 | -0.001 | -0.006 | -0.000 |
|  |  | (0.002) | (2.989) | (0.001) | (0.006) | (0.002) |
|  | WB avg 10-15 | -0.000 | -1.068 | 0.001 | 0.001 | 0.001 |
|  |  | (0.001) | (2.916) | (0.001) | (0.004) | (0.001) |
|  | WB avg 20-25 | 0.000 | -0.427 | 0.000 | -0.001 | 0.000 |
|  |  | (0.000) | (0.714) | (0.000) | (0.002) | (0.001) |
|  | Wet bulb avg 25+ | 0.001 | -0.818 | 0.000 | 0.000 | 0.001** |
|  |  | (0.001) | (0.788) | (0.000) | (0.003) | (0.001) |
|  |  |  |  |  |  |  |
|  |  |  |  |  |  |  |
| Constant | | 0.132** | 3,393.610*** | 0.096* | 8.522*** | 0.123 |
|  |  | (0.063) | (108.642) | (0.057) | (0.341) | (0.096) |
|  |  |  |  |  |  |  |
| N |  | 34,258 | 34,258 | 34,258 | 34,258 | 34,258 |
| R-sq |  | 0.085 | 0.125 | 0.087 | 0.081 | 0.088 |

This table shows the regression coefficients and cluster-robust standard errors in parentheses from the model specified in equation (1) using the wet bulb heat metric. Estimates are shown for preterm birth and four other measures of health at birth. As specified in equation (1), the regressions also include covariates (mother’s age, Aboriginal status, whether mother’s first pregnancy), month-year fixed effects and location-month-sex fixed effects (these are absorbed using the Stata ‘areg’, which affects the intercept but not the coefficients).
