# Supplementary material for: The relationship between prenatal heat exposure and birth outcomes: How much does the heat metric matter?
Source: PLoS One. 2025 Sep 3;20(9):e0330498. doi: 10.1371/journal.pone.0330498 (PMC12407402; doi:10.1371/journal.pone.0330498)
Supplement: S9 Table — (DOCX) [file pone.0330498.s014.docx]

**S9 Table: average wet bulb piecewise linear with additional outcomes**

|  |  | Preterm birth | Birthweight (grams) | Small for Gestational Age | Apgar 5 score | Special Care Nursery |
| --- | --- | --- | --- | --- | --- | --- |
|  | WB avg <10 | -0.000 | 0.745 | -0.001 | 0.001 | -0.001* |
| 1st tri |  | (0.000) | (0.978) | (0.001) | (0.002) | (0.001) |
|  | WB avg 10-15 | 0.000 | -0.495 | 0.000 | 0.004 | -0.000 |
|  |  | (0.001) | (0.909) | (0.001) | (0.002) | (0.001) |
|  | WB avg 20-25 | 0.000 | -0.633 | -0.000 | 0.000 | -0.000 |
|  |  | (0.001) | (0.556) | (0.000) | (0.001) | (0.001) |
|  | WB avg 25+ | -0.000 | 0.139 | -0.001** | 0.002 | -0.000 |
|  |  | (0.000) | (0.459) | (0.000) | (0.001) | (0.000) |
|  | WB avg <10 | 0.000 | 0.694 | 0.000 | 0.001 | -0.001 |
|  |  | (0.000) | (0.500) | (0.001) | (0.002) | (0.001) |
| 2nd tri | WB avg 10-15 | -0.001* | 0.283 | -0.000 | 0.001 | -0.001 |
|  |  | (0.001) | (1.312) | (0.001) | (0.003) | (0.001) |
|  | WB avg 20-25 | -0.001** | -0.388 | -0.000 | 0.001 | -0.001 |
|  |  | (0.000) | (0.885) | (0.000) | (0.001) | (0.001) |
|  | WB avg 25+ | -0.000 | -0.567 | -0.000 | 0.001 | -0.001* |
|  |  | (0.000) | (0.661) | (0.000) | (0.002) | (0.000) |
|  | WB avg <10 | -0.001 | 1.519** | -0.000 | 0.001 | -0.001 |
|  |  | (0.001) | (0.672) | (0.000) | (0.003) | (0.000) |
|  | WB avg 10-15 | 0.001 | -1.161 | 0.001 | -0.003 | 0.000 |
|  |  | (0.001) | (1.578) | (0.001) | (0.005) | (0.001) |
|  | WB avg 20-25 | 0.000 | -0.198 | 0.000 | 0.001 | 0.000 |
| 3rd tri |  | (0.000) | (0.737) | (0.000) | (0.002) | (0.001) |
|  | WB avg 25+ | 0.000 | -0.409 | 0.000 | 0.003 | 0.000 |
|  |  | (0.001) | (0.761) | (0.000) | (0.003) | (0.001) |
|  |  |  |  |  |  |  |
|  |  |  |  |  |  |  |
|  | Constant | 0.110* | 3,368.704*** | 0.122*** | 8.644*** | 0.156 |
|  |  | (0.058) | (120.327) | (0.043) | (0.279) | (0.102) |
|  | N |  |  |  |  |  |
|  |  | 34,258 | 34,258 | 34,258 | 34,258 | 34,258 |
|  | R-sq | 0.084 | 0.124 | 0.087 | 0.080 | 0.087 |

This table shows the regression coefficients and cluster-robust standard errors in parentheses from the model specified in equation (1) using counts of the number of days with average wet bulb temperature under 10, 10-15, 20-25, and 25+. Estimates are shown for preterm birth and four other measures of health at birth. As specified in equation (1), the regressions also include covariates (mother’s age, Aboriginal status, whether mother’s first pregnancy), month-year fixed effects and location-month-sex fixed effects (these are absorbed using the Stata ‘areg’, which affects the intercept but not the coefficients).
