# Supplementary material for: The relationship between prenatal heat exposure and birth outcomes: How much does the heat metric matter?
Source: PLoS One. 2025 Sep 3;20(9):e0330498. doi: 10.1371/journal.pone.0330498 (PMC12407402; doi:10.1371/journal.pone.0330498)
Supplement: S10 Table — (DOCX) [file pone.0330498.s015.docx]

**S10 Table: maximum wet bulb (WB) piecewise specification with additional outcomes**

|  |  | Preterm birth | Birthweight (grams) | Small for Gestational Age | Apgar 5 score | Special Care Nursery |
| --- | --- | --- | --- | --- | --- | --- |
|  | WB max <10 | 0.001 | 4.252 | -0.002 | 0.007 | -0.000 |
| 1st tri |  | (0.002) | (3.658) | (0.002) | (0.006) | (0.001) |
|  | WB max 10-15 | -0.001* | 1.709* | -0.000 | 0.002 | -0.002* |
|  |  | (0.001) | (0.917) | (0.001) | (0.003) | (0.001) |
|  | WB max 15-20 | -0.000 | 0.971 | -0.000 | 0.005*** | -0.001** |
|  |  | (0.001) | (0.628) | (0.001) | (0.001) | (0.000) |
|  | WB max 25-30 | -0.000 | 0.324 | -0.000 | 0.001 | 0.000 |
|  |  | (0.000) | (0.341) | (0.000) | (0.001) | (0.000) |
|  | WB max 30+ | -0.000 | 0.682 | 0.000 | 0.001 | -0.000 |
|  |  | (0.000) | (0.829) | (0.001) | (0.002) | (0.001) |
| 2nd tri | WB max <10 | -0.000 | -1.020 | -0.000 | -0.013** | -0.003*** |
|  |  | (0.001) | (2.534) | (0.001) | (0.005) | (0.001) |
|  | WB max 10-15 | 0.000 | 1.534 | 0.000 | 0.003 | -0.001 |
|  |  | (0.001) | (0.930) | (0.001) | (0.003) | (0.001) |
|  | WB max 15-20 | -0.000 | 0.259 | -0.000 | -0.001 | -0.000 |
|  |  | (0.000) | (0.707) | (0.000) | (0.002) | (0.000) |
|  | WB max 25-30 | -0.000 | -0.661** | 0.000 | 0.000 | -0.000 |
|  |  | (0.000) | (0.333) | (0.000) | (0.001) | (0.000) |
|  | WB max 30+ | 0.000 | -0.124 | 0.001*** | 0.000 | 0.001** |
|  |  | (0.000) | (0.512) | (0.000) | (0.002) | (0.000) |
| 3^rd^ tri | WB max <10 | 0.002 | -1.774 | -0.000 | -0.004 | 0.002* |
|  |  | (0.001) | (1.598) | (0.002) | (0.006) | (0.001) |
|  | WB max 10-15 | -0.002*** | 2.683*** | -0.000 | 0.004 | -0.001* |
|  |  | (0.001) | (0.873) | (0.000) | (0.003) | (0.001) |
|  | WB max 15-20 | 0.001 | -0.736 | 0.000 | -0.004 | -0.000 |
|  |  | (0.001) | (0.714) | (0.000) | (0.003) | (0.000) |
|  | WB max 25-30 | -0.000 | -0.092 | 0.000* | 0.003* | 0.000 |
|  |  | (0.000) | (0.359) | (0.000) | (0.001) | (0.000) |
|  | WB max 30+ | 0.001 | -1.243 | 0.000 | 0.000 | 0.001 |
|  |  | (0.001) | (1.871) | (0.000) | (0.003) | (0.001) |
|  |  |  |  |  |  |  |
|  |  | 0.100*** | 3,331.013*** | 0.090*** | 8.717*** | 0.117*** |
| Constant |  | (0.027) | (44.384) | (0.026) | (0.132) | (0.027) |
|  |  |  |  |  |  |  |
| N |  | 34,258 | 34,258 | 34,258 | 34,258 | 34,258 |
| R-sq |  | 0.085 | 0.125 | 0.087 | 0.080 | 0.087 |
|  |  |  |  |  |  |  |

This table shows the regression coefficients and cluster-robust standard errors in parentheses from the model specified in equation (1) using the counts of the number of days with maximum wet bulb temperatures under 10, 10-15, 15-20, 25-30, and 30+. We estimate maximum wet bulb temperatures using Stull’s (2011) equation based on maximum air temperature and humidity. Estimates are shown for preterm birth and four other measures of health at birth. As specified in equation (1), the regressions also include covariates (mother’s age, Aboriginal status, whether mother’s first pregnancy), month-year fixed effects and location-month-sex fixed effects (these are absorbed using the Stata ‘areg’, which affects the intercept but not the coefficients).
