# Supplementary material for: The relationship between prenatal heat exposure and birth outcomes: How much does the heat metric matter?
Source: PLoS One. 2025 Sep 3;20(9):e0330498. doi: 10.1371/journal.pone.0330498 (PMC12407402; doi:10.1371/journal.pone.0330498)
Supplement: S11 Table — (DOCX) [file pone.0330498.s016.docx]

**S11 Table: Regression coefficient estimates by climate zone – air temperature**

|  |  | Tropical | Arid | Tropical | Arid |
| --- | --- | --- | --- | --- | --- |
| 1^st^ tri | Max <20 | 0.014 | 0.002 | 0.021 | 0.003** |
|  |  | (0.027) | (0.002) | (0.023) | (0.002) |
|  | Max 20-25 | 0.001** | 0.002** | 0.002** | 0.004** |
|  |  | (0.001) | (0.001) | (0.001) | (0.002) |
|  | Max 30-35 | 0.000 | 0.001 | 0.000 | 0.001 |
|  |  | (0.000) | (0.001) | (0.000) | (0.001) |
|  | Max 35-40 | 0.000 | 0.003*** | 0.000 | 0.003* |
|  |  | (0.000) | (0.001) | (0.000) | (0.002) |
|  | Max 40+ | -0.000 | 0.004*** | -0.000 | 0.002 |
|  |  | (0.001) | (0.001) | (0.001) | (0.002) |
|  | Min <5 |  |  | -0.007* | 0.001 |
|  |  |  |  | (0.004) | (0.002) |
|  | Min 5-10 |  |  | -0.004* | -0.001 |
|  |  |  |  | (0.002) | (0.002) |
|  | Min 10-15 |  |  | 0.000 | 0.001 |
|  |  |  |  | (0.001) | (0.002) |
|  | Min 20-25 |  |  | 0.001*** | 0.001 |
|  |  |  |  | (0.000) | (0.001) |
|  | Min 25+ |  |  | 0.001*** | 0.003 |
|  |  |  |  | (0.000) | (0.002) |
|  | Max <20 | 0.015 | -71.278*** | 0.013 | -0.002 |
| 2^nd^ tri |  | (0.023) | (18.577) | (0.030) | (0.002) |
|  | Max 20-25 | -0.002*** | 3.097* | -0.003*** | -0.001 |
|  |  | (0.001) | (1.618) | (0.001) | (0.002) |
|  | Max 30-35 | -0.000 | -0.596* | 0.000 | 0.002 |
|  |  | (0.000) | (0.325) | (0.000) | (0.002) |
|  | Max 35-40 | -0.000 | 0.168 | 0.001 | 0.001 |
|  |  | (0.000) | (0.296) | (0.000) | (0.002) |
|  | Max 40+ | -0.000 | -1.283 | -0.000 | 0.002 |
|  |  | (0.001) | (1.155) | (0.001) | (0.003) |
|  | Min <5 |  |  | -0.090* | 0.004* |
|  |  |  |  | (0.047) | (0.003) |
|  | Min 5-10 |  |  | 0.000 | 0.003 |
|  |  |  |  | (0.001) | (0.003) |
|  | Min 10-15 |  |  | 0.000 | 0.005** |
|  |  |  |  | (0.001) | (0.002) |
|  | Min 20-25 |  |  | 0.001** | 0.003 |
|  |  |  |  | (0.000) | (0.003) |
|  | Min 25+ |  |  | 0.001*** | 0.002 |
|  |  |  |  | (0.000) | (0.003) |
| 3^rd^ tri | Max <20 | -0.008 | 43.241 | -0.027 | -0.004 |
|  |  | (0.011) | (36.817) | (0.018) | (0.006) |
|  | Max 20-25 | -0.002 | 2.920 | -0.003 | -0.001 |
|  |  | (0.002) | (2.822) | (0.002) | (0.003) |
|  | Max 30-35 | 0.000** | -0.469 | 0.000*** | 0.004*** |
|  |  | (0.000) | (0.316) | (0.000) | (0.001) |
|  | Max 35-40 | 0.000 | -0.401 | 0.001*** | 0.007*** |
|  |  | (0.000) | (0.474) | (0.000) | (0.002) |
|  | Max 40+ | 0.003*** | -2.746* | 0.003*** | 0.008*** |
|  |  | (0.001) | (1.634) | (0.001) | (0.002) |
|  | Min <5 |  |  | 0.019 | 0.004 |
|  |  |  |  | (0.020) | (0.005) |
|  | Min 5-10 |  |  | 0.003 | 0.003 |
|  |  |  |  | (0.003) | (0.003) |
|  | Min 10-15 |  |  | 0.000 | 0.006*** |
|  |  |  |  | (0.001) | (0.002) |
|  | Min 20-25 |  |  | 0.000 | -0.002** |
|  |  |  |  | (0.000) | (0.001) |
|  | Min 25+ |  |  | 0.000 | -0.003* |
|  |  |  |  | (0.000) | (0.002) |
|  |  |  |  |  |  |
|  |  |  |  |  |  |
| Constant | | 0.076*** | 0.070*** | 0.025 | -0.168*** |
|  |  | (0.020) | (0.189) | (0.051) | (0.253) |
|  |  |  |  |  |  |
| N |  | 26,521 | 7,379 | 26,521 | 7,379 |
| R-sq |  | 0.069 | 0.170 | 0.070 | 0.179 |

This table shows the regression coefficients and cluster-robust standard errors in parentheses from the model specified in equation (1) using both benchmark heat metric and the Max + Min heat metric, with the sample split by climate zone Estimates are shown for preterm birth and four other measures of health at birth. As specified in equation (1), the regressions also include covariates (mother’s age, Aboriginal status, whether mother’s first pregnancy), month-year fixed effects and location-month-sex fixed effects (these are absorbed using the Stata ‘areg’, which affects the intercept but not the coefficients).
