# Supplementary material for: The relationship between prenatal heat exposure and birth outcomes: How much does the heat metric matter?
Source: PLoS One. 2025 Sep 3;20(9):e0330498. doi: 10.1371/journal.pone.0330498 (PMC12407402; doi:10.1371/journal.pone.0330498)
Supplement: S13 Table — (DOCX) [file pone.0330498.s018.docx]

**S13 Table: Measures of model fit for each heat metric – tropical climate zone**

|  | P-val: joint significance of heat exposure terms | Adj R^2^ | AIC |
| --- | --- | --- | --- |
| **Panel A: Tropical climate zone** | | | |
| Benchmark | 0.00 | 0.031 | 10240 |
| Trimester average | 0.00 | 0.031 | 10265 |
| Heatwave count | 0.00 | 0.031 | 10261 |
| **Max and min** | **0.00** | **0.032** | **10207** |
| Wet bulb | 0.00 | 0.030 | 10244 |
| **Panel B: Arid climate zone** | | | |
| Benchmark | 0.00 | 0.058 | 2477 |
| Trimester average | 0.00 | 0.055 | 2509 |
| Heatwave count | 0.00 | 0.051 | 2540 |
| **Max and min** | **0.00** | **0.065** | **2401** |
| Wet bulb | 0.00 | 0.056 | 2472 |

Each row of this table represents results from a regression using the indicated heat metric, for the sample in tropical climate zones only in panel A and the sample in arid climate zones only in panel B. The first column in this table shows p-values for an F-test of joint statistical significance of all terms in the heat exposure metric. We present adjusted R-squared as this penalises additional coefficients, and some metrics (Max and min and Wet bulb) have many more coefficients than the other metrics. The Akaike Information Criteria (AIC) is a measure of model fit suitable for comparing across models with different coefficients but the same outcome measure. A lower value indicates better fit. Sample size: 26,521 tropical climate zone and 7,379 arid climate zone.
