# Supplementary material for: The relationship between prenatal heat exposure and birth outcomes: How much does the heat metric matter?
Source: PLoS One. 2025 Sep 3;20(9):e0330498. doi: 10.1371/journal.pone.0330498 (PMC12407402; doi:10.1371/journal.pone.0330498)
Supplement: S14 Table — (DOCX) [file pone.0330498.s019.docx]

## **S14 Table: Correlations coefficients among various weather metrics in Darwin**

|  | Rainfall (ml) | Average wet bulb temperature | Maximum air temperature | Relative humidity (%) |
| --- | --- | --- | --- | --- |
| Rainfall (ml) | 1.0000 |  |  |  |
| Average wet bulb temperature | 0.3180 | 1.0000 |  |  |
| Maximum air temperature | –0.3268 | 0.2185 | 1.0000 |  |
| Relative humidity (%) | 0.4782 | 0.8059 | –0.3531 | 1.0000 |

Daily NASA reanalysis data from Darwin, January 2020 to December 2023
