# Supplementary material for: The relationship between prenatal heat exposure and birth outcomes: How much does the heat metric matter?
Source: PLoS One. 2025 Sep 3;20(9):e0330498. doi: 10.1371/journal.pone.0330498 (PMC12407402; doi:10.1371/journal.pone.0330498)
Supplement: Attachment — Submitted filename: Referee_report_PONE-D-25-13859-20250515195052.pdf [file pone.0330498.s020.pdf]

- Manuscript Number: PONE-D-25-13859
- Title: The relationship between prenatal heat exposure and birth outcomes: how much does
the heat metric matter?

- **Overview and general recommendation**

This paper studies the impact of heat exposure on preterm birth using administrative data from the Northern Territory of Australia. It highlights the importance of heat metrics on the estimation results and presents an exercise in the metrics selection procedure. I find this paper's findings potentially important, and its perspective on the measurement issue is innovative. The paper is also well written and has the potential to make a significant contribution to the environmental econometrics literature.

I have some concerns regarding the methodology and research design for the editor to consider. I elaborate on my comments and suggestions below:

- **Major comments**

1. **Criteria for selecting preferred model selection.**

The main potential contribution of this paper is to provide a model selection approach among the commonly used heat exposure metrics. Table 2 presents the most important results. However, I am somewhat reluctant to agree with the three selection criteria that the authors use. They employ a two-step selection method: first, they exclude the inferior candidates using Joint F-statistics; then, they select the best one based on Adjusted R^2 and AIC. I have three comments.

Econometrically Distinguishable? The first issue is that the adjusted R^2 and AIC for alternative metrics are too close to strongly support which one is better. For the adjusted R^2, all five metrics fall between 0.035 and 0.037. I would expect a much larger difference in R^2, given that Figure 1 shows that in your studied context, the daily maximum and minimum temperatures are not correlative. I would anticipate that the five candidates could explain a significant amount of variation.

Additionally, this absolute magnitude is surprisingly small, especially considering that the authors have controlled for rich fixed effects. This may indicate that other factors are interpreting the majority of the variation in the data. More importantly, if you include some of these variables, would the current ranking persist?

Apple-to-Apple Comparison? The second issue is that I am not sure if the five metrics are comparable in an Apple-to-Apple manner, and whether it is fair to compare them directly. Based on the econometric specifications, Benchmark, Max, Min, and Wet Bulb seem to be comparable with the other two metrics. As a researcher who also works on heat exposure, I prefer the band specification over averages and counts, unless the data quality does not allow for this. Therefore, it is not usually an either-or question.

Is a high goodness of fit indicative of a better heat metric model? The third issue is that I am uncertain whether a higher goodness of fit indicates a better heat metric model. A higher goodness of fit metric explains the data variation better but does not necessarily capture the underlying mechanisms more effectively. I would imagine that some data-driven approaches, like Lasso and neural networks, would yield a much better goodness of fit, but they may not be preferred in research. The discussion regarding this issue is crucial to justify the methodology.

2. Connecting the theoretical channel to empirical results and interpretation of key parameters.

The three theoretical channels that the paper proposes and the empirical pattern do not seem closely tied. For example, from "Exposure to cooler temperatures that contribute to lower risk" to show the prolonged heat exposure to heat strain seems quite speculative to me. I am also not sure how you could test for the "Maternal behaviour" channel without data on factors such as labor participation and the performance of mothers.

My interpretation of Figure 4 would be quite different: overall null effect on heat exposure from the 1st and 2nd trimesters; substantial and close-to-linear effects for the 3rd trimester. I would dig more into the heterogeneity across trimesters.

3. Should one category being omitted in this research design?

Could you elaborate on why it would be necessary to omit one category for the band specifications? In the literature, this is commonly done because the total sum of each band is consistent for everyone, such as 365 days or 24 hours. However, in your case, pregnancy duration clearly varies for each baby. Unless you account for this in X_i, it seems there might be a missing effect from the overall duration of pregnancy.

4. **The use of fixed effect model**

By including the location-month-gender FE, the model is essentially comparing boys (girls) born in the same month and location. I would expect these fixed effects to absorb much variation that you would like to use for identification. Can you explain, e.g., using an example, what you are comparing conditional on these fixed effects?

Also, can your data track the location of the mother over the pregnancy cycle? Or are you assuming the location of the mother does not change? It seems to be an important assumption to explicitly discuss for the fixed effect model to control for omitted variable bias, such as potential sorting on place of residence and timing of conception.

5. Sample period.

Page 15, lines 298-301, the paper states: the administrative data is extracted from January 1994 to 31 December 2014, but the regression sample is from March 2000 to September 2009. I did not understand the justification behind this sample period selection.

- **Minor comments**

1. Page 11, line 213: The font style of channel “Maternal behavioral” should be the same as the previous two channels.
2. Figure 4, 7, 8: I recommend the authors consider breaking the coefficient for each trimester into a separate subfigure. The current deployment of the coefficient is quite messy and hard to interpret.
3. Page 23, line 446, equation (1): I believe the usage of subscript itj for preterm_{itj} might be inappropriate as it could confuse people into thinking the data is individual-level panel data. For each baby i, there is no variation at t and j. I suggest just using preterm_{i} or preterm_{i(tj)}.
4. Why does Figure 1 use the data from 2020-2023 while the main regression uses 2000 to 2009?
5. In the first sentence of the abstract: “The impact of prenatal heat exposure on birth outcomes is well-established, but what is it about heat that affects prenatal development?” It is not clear to me why heat that affects prenatal development has to do with the main goal of the paper.
6. Adding citation if you would like to speak more to the literature on environmental economics methodology literature.

Ghanem, D., & Smith, A. (2021). What are the benefits of high-frequency data for fixed effects panel models?. Journal of the Association of Environmental and Resource Economists, 8(2), 199-234.
