# Supplementary material for: The relationship between prenatal heat exposure and birth outcomes: How much does the heat metric matter?
Source: PLoS One. 2025 Sep 3;20(9):e0330498. doi: 10.1371/journal.pone.0330498 (PMC12407402; doi:10.1371/journal.pone.0330498)
Supplement: Attachment — Submitted filename: Response to reviewers.docx [file pone.0330498.s022.docx]

# Editor

Both reviewers appreciate the importance of your research questions and find merits in your general approach. In addition to the general comments, Reviewer #1 suggested alternative, machine-learning-based approaches to the variable selection problem (e.g. lasso), which is standard in the recent econometrics/statistics literature and can be reconciled with a causal interpretation (see work by Susan Athey, Victor Chernozhukov, et al.) I agree with the reviewer and think there are merits in trying that.

Reviewer #1 also suggested a band-based approach standard in the climate impact literature, which can be combined with a wet-bulb-based temperature metric (see e.g., Melissa Lapalo, 2023 AEJ: Applied). I'll leave that to you as to whether you want to implement such an approach.

While revising your manuscript, please consider all issues mentioned in the reviewers' comments carefully: please outline every change made in response to their comments and provide suitable rebuttals for any comments not addressed. Please note that I intend to go back to the referees for additional feedback.

Thank you very much. We will respond to each point below, with the edits we’ve made to the text and references to page numbers – note that these are page numbers **in the track changes version of the manuscript.**

We will respond to those specific suggestions from Reviewer 1 below – as described below, we did consider machine learning methods at length and have now added an explanation in the manuscript outlining this.

We hadn’t seen the paper by Melissa LoPalo but this is interesting as an example of when wet bulb temperatures are actually more relevant than dry bulb temperature, thank you for sharing this. In fact in our analysis we do use the same metric – wet bulb temperature split into bands (this is our ‘wet bulb’ metric). And in this paper LoPalo uses the same type of reanalysis data as we do. So it indicates that it’s not necessarily poorer quality reanalysis data for wet bulb measures that prevents us from detecting an effect in our analysis – it may be that wet bulb affects productivity/behaviour while dry bulb temperatures have more direct effects on health – though this is speculative. We have added the following sentence to our discussion making this point broadly, but haven’t gone into any more speculation on what kinds of outcomes wet bulb vs dry temperatures are better suited to.

*“That said, we note that studies using reanalysis wet bulb data for other outcomes, such as LoPalo’s (2023) analysis of the impacts of heat on worker productivity, do find wet bulb measures to be more predictive than ambient temperatures.” [pg 41]*

# Reviewer 1

Overview and general recommendation
This paper studies the impact of heat exposure on preterm birth using administrative data from the Northern Territory of Australia. It highlights the importance of heat metrics on the estimation results and presents an exercise in the metrics selection procedure. I find this paper's findings potentially important, and its perspective on the measurement issue is innovative. The paper is also well written and has the potential to make a significant contribution to the environmental econometrics literature.

Thank you for your helpful feedback.

I have some concerns regarding the methodology and research design for the editor to consider. I elaborate on my comments and suggestions below:

Major comments
**1.Criteria for selecting preferred model selection.**
The main potential contribution of this paper is to provide a model selection approach among the commonly used heat exposure metrics. Table 2 presents the most important results. However, I am somewhat reluctant to agree with the three selection criteria that the authors use. They employ a two-step selection method: first, they exclude the inferior candidates using Joint F-statistics; then, they select the best one based on Adjusted R² and AIC. I have three comments.

Econometrically Distinguishable?

The first issue is that the adjusted R² and AIC for alternative metrics are too close to strongly support which one is better. For the adjusted R², all five metrics fall between 0.035 and 0.037. I would expect a much larger difference in R², given that Figure 1 shows that in your studied context, the daily maximum and minimum temperatures are not correlative. I would anticipate that the five candidates could explain a significant amount of variation.
Additionally, this absolute magnitude is surprisingly small, especially considering that the authors have controlled for rich fixed effects. This may indicate that other factors are interpreting the majority of the variation in the data. More importantly, if you include some of these variables, would the current ranking persist?

Thank you for these thoughtful reflections. Yes we agree that the differences between the R-sq are small, and concluding that one model is better than another just because the adjusted R-sq is 0.037 vs 0.036, is reading too much into it. This is why we put a lot of thought into the conceptual framework. Ultimately, while in this case we find the ‘max and min’ metric fits marginally better, we see the combination of conceptual framework and case study as the value of this paper. That’s why, based on our conceptual framework, we state in the discussion (Section 5.2) that the ‘trimester average’ and ‘heatwave count’ metrics are unlikely reflect reality.

On the reasons for such low R-squared figures – there are so many factors that can influence the risk of preterm birth outcomes, of which heat exposure is one small contributor. For an individual, the increased risk of being born preterm because of a marginal increase in heat exposure in pregnancy is tiny – there are so many more proximate factors. But despite that, we (and many others) believe understanding the impacts of heat exposure on birth outcomes is important because at the population level – especially in a context with high and increasing average heat exposure – these effects add up.

We have added the following to our discussion:

*“While we do find one metric that outperforms the alternatives on model fit, the differences appear marginal – our preferred ‘max and min’ metric has an adjusted R-squared that is just 0.001 units higher than the benchmark metric. Furthermore, the explanatory power of even our preferred heat metric is low – clearly there are many more proximate factors, not included in our models, that explain the vast majority of variation in preterm birth rates. That said, while the effects of prenatal heat exposure are small for any one individual, these impacts are important at the population level. This is particularly so in the context of high and rising levels of heat exposure”. [pg 38]*

On the exact level of the R-squared – this does not incorporate the explanatory power of the full model, because the community x sex x month FEs are absorbed and not estimated (in Stata we use the command ‘areg’ – which gives us the same estimates on our regressors of interest as if we had estimated the FEs directly, but is much less computationally intensive, which is important given we estimate many version of the same model). If instead we were to run models that directly estimate those FEs, the R-squared would be closer to 0.09 (see table below).

In the table below, we have also tested whether the same ranking persists when we include additional covariates – they do. The set of potential covariates is limited in our data, but we have included: an indicator for whether there were any obstetric complications, whether the mother has any complications in her medical history, the number of antenatal visits the mother attended, and whether the mother reported drinking alcohol at her first antenatal visit. We did not include these variables in our main regression due to missing data and concerns over data reliability/quality and confounding – though they are useful as a test for whether the goodness of fit ranking remains.

|  | Absorbed regressors (as in main analysis) | | Directly estimated FEs | | Absorbed regressors with additional covariates* | |
| --- | --- | --- | --- | --- | --- | --- |
|  | Adjusted R sq | AIC | Adjusted R sq | AIC | Adj R-sq | AIC |
| Benchmark metric | 0.036 | 13214 | 0.086 | 13218 | 0.083 | 6014 |
| Trimester average | 0.035 | 13251 | 0.085 | 13259 | 0.082 | 6050 |
| Heatwave count | 0.035 | 13267 | 0.084 | 13273 | 0.081 | 6062 |
| Max and min (preferred) | 0.037 | 13174 | 0.087 | 13178 | 0.083 | 5985 |
| Wet bulb | 0.035 | 13233 | 0.085 | 13233 | 0.082 | 6029 |

* number of antenatal visits, whether mother has pre-existing medical conditions, whether any obstetric complications, whether mother reported drinking alcohol at first antenatal visit.

Apple-to-Apple Comparison?

The second issue is that I am not sure if the five metrics are comparable in an Apple-to-Apple manner, and whether it is fair to compare them directly. Based on the econometric specifications, Benchmark, Max, Min, and Wet Bulb seem to be comparable with the other two metrics. As a researcher who also works on heat exposure, I prefer the band specification over averages and counts, unless the data quality does not allow for this. Therefore, it is not usually an either-or question.

In our analysis of goodness of fit, we have done our best to make these technically comparable using AIC (which is not sensitive to number of regressors) and an adjusted R-sq (which penalises models with more regressors) instead of standard R-sq. But you are right that in a practical sense, researchers working in this field might choose one metric because others are not available.^[1]^ However, we hope this paper will also be useful for researchers who are new to thinking about heat exposure. In fact – the reason we did this analysis initially was to decide for ourselves which metric to use in subsequent work, because we did not find any concrete guidance.

To clarify this, we have added to section 5.2 in our discussion of the Trimester average and Heatwave count metrics:

*“Therefore, where researchers have the choice, we recommend against using the trimester average or heatwave count metrics.” [pg 41]*

Is a high goodness of fit indicative of a better heat metric model?

The third issue is that I am uncertain whether a higher goodness of fit indicates a better heat metric model. A higher goodness of fit metric explains the data variation better but does not necessarily capture the underlying mechanisms more effectively. I would imagine that some data-driven approaches, like Lasso and neural networks, would yield a much better goodness of fit, but they may not be preferred in research. The discussion regarding this issue is crucial to justify the methodology.

This is a helpful prompt – thank you. And again, this is why we see the conceptual framework as a key part of the paper. Our discussion and conclusion draw on both our empirical findings and the concepts. But you are right that if, for instance, we had found that the heatwave metric fitted the data best, we probably still wouldn’t want to recommend using that metric alone in future research.

On machine learning – in fact we did initially set out planning to use machine learning. We ran analysis using a Lasso regression, which allowed us to include covariates representing all of the heat metrics we test, and for the Lasso algorithm to select the covariates that best fit the data. We had hoped that this approach would help us to identify which metric/s to use. However, we ultimately decided that ML was not appropriate for this particular case. Contrary to our (and your) expectations, the Lasso models did not yield a meaningfully higher goodness of fit. Our interpretation of this comes back to the point above about how low the R-squared is – the explanatory power of various metrics of heat exposure in predicting whether a baby is born preterm or not is tiny.

When we applied Lasso regression, this led to the regularisation problem: that is, because the explanatory power of heat exposure on birth outcomes is relatively small—as is typical in environmental health research where a single environmental factor might explain only modest portions of variation—the Lasso penalty systematically eliminated heat exposure variables in favour of models with no heat predictors at all. This occurred not because heat exposure lacks causal importance, but because the penalty structure prioritises parsimony over detecting small but meaningful effects.

We are aware that double/debiased machine learning approaches can address this regularisation bias – e.g. Chernozhukov et al 2018 ([link](https://academic.oup.com/ectj/article-abstract/21/1/C1/5056401?redirectedFrom=fulltext)). But this process led us to conclude that we would see little value add from ML approaches. Given the very small explanatory power of any heat exposure metric on our outcomes of interest, and the small differences in fit amongst the metrics, we think a double ML approach would likely still struggle to discriminate between metrics. Hence we decided that the best way to answer our research question – and convey this answer to a broad audience – was to stick with the simpler ‘horserace’ regression approach.

But thank you for raising this because none of this thinking is included in the manuscript, and it should be. Reflecting on this, we have added a short section to explain the reasons we decided against using machine learning in the manuscript.

### Machine learning considerations

“In this analysis, we set out to select a preferred heat metric from among many potential regressors. To do this, we initially considered use of machine learning – and in particular lasso regression. However, in this case we determined that a machine learning approach was not appropriate because of the low explanatory power of heat exposure variables relative to other factors that determine the risk of preterm birth. As we show in our analysis and address in our discussion, heat exposure explains a very small share of variation in outcomes. The differences in explanatory power between alternative metrics are smaller still (see Setion 5.1 below for a discussion). Hence, when we apply a lasso regression, the penalty structure prioritises parsimony over detecting small but meaningful effects – and eliminates heat exposure variables. We are aware that double machine learning approaches can help to address the regularisation bias. But we determined that, given the goal of our analysis was to make general recommendations for how heat exposure should be measured, we favoured a more direct approach to comparing alternative metrics”. [pp25-26]

**2.Connecting the theoretical channel to empirical results and interpretation of key parameters.**

The three theoretical channels that the paper proposes and the empirical pattern do not seem closely tied. For example, from “Exposure to cooler temperatures that contribute to lower risk” to show the prolonged heat exposure to heat strain seems quite speculative to me. I am also not sure how you could test for the “Maternal behaviour” channel without data on factors such as labor participation and the performance of mothers.

My interpretation of Figure 4 would be quite different: overall null effect on heat exposure from the 1st and 2nd trimesters; substantial and close-to-linear effects for the 3rd trimester. I would dig more into the heterogeneity across trimesters.

Yes we completely agree it is not possible to test for the impact of maternal behaviour in the data we have. We think it is worth including in our diagram as behaviour is a key mechanism in linking prenatal heat exposure to outcomes for the baby, both as an effect modifier for how heat leads to heat stress and heat strain, and through other channels as well. But it is not something we can observe.

On exposure to cooler temperatures – thank you, we have toned down this language. However, the point we want to make is that heat exposure can affect the baby in utero even if it is not extreme heat. The incorporation of ‘prolonged’ however, is drawing on the context – where it is almost always hot in the NT, hence prolonged exposure. But you are right that daily maximum temperature provides no evidence on the duration of exposure beyond a single day, and we don’t have a good sense of how long exposure needs to be to lead to heat strain (possibly a day, though longer exposure may also be relevant). Hence this connection between the evidence, context and theories fits better in the discussion and conclusion, not in the results section.

On figure 4 – the estimates are small, but we think still meaningful given the small units of regressors – i.e. it’s just one additional day within each band, within a roughly 3 month period. In terms of whether heat exposure in the first and second trimesters are overall null, we have produced the below table to help us consider. It shows results from F-tests of the joint statistical significance of regressors in the benchmark model, by trimester. The regressors for the benchmark metric (which is the one shown in Fig 4) are jointly significant at the 5% level for trimesters 2 and 3, but not for trimester 1.

|  | Benchmark | | Max + min (preferred) | |
| --- | --- | --- | --- | --- |
|  | F stat | P val | F stat | P val |
| Trimester 1 regressors | 1.92 | 0.0970 | 3.56 | 0.0004 |
| Trimester 2 regressors | 3.75 | 0.0036 | 4.93 | 0.0000 |
| Trimester 3 regressors | 4.00 | 0.0023 | 4.97 | 0.0000 |

We have added the following text to page 13, Section 2.3 (on the implications of our conceptual framework for heat exposure measurement):

*“However, our conceptual framework also points out a potential moderator between heat exposure and birth outcomes that we are unable to measure in our data – maternal behaviour. While maternal behavioural responses to heat exposure do not feature in our data analysis, behaviour is likely a key factor, and may help to explain differences in the magnitudes and timing of effects (in terms of whether exposure has different effects depending on the timing of exposure during pregnancy) across different populations and contexts.” [pg 13]*

And changed our summary of Figure 4, from:

*‘We see that exposure to temperatures between 30 and 40 degrees in the first trimester slightly increases the risk of preterm birth’*

To:

*‘We can see little impact from heat exposure in the first trimester.’*

**3.Should one category being omitted in this research design?**
Could you elaborate on why it would be necessary to omit one category for the band specifications? In the literature, this is commonly done because the total sum of each band is consistent for everyone, such as 365 days or 24 hours. However, in your case, pregnancy duration clearly varies for each baby. Unless you account for this in X_i, it seems there might be a missing effect from the overall duration of pregnancy.

Good point, and this again raises a methodological detail that was not evident in the manuscript, and we have now added it in. The way we have constructed the heat exposure variables is working 39 weeks forward from the date of conception, meaning that exposures are measured through 39 weeks’ gestation (which is the average in the data), even for those who were born before 39 weeks.

We thought a lot about this, because clearly where our outcome is preterm birth, this means that those experiencing preterm birth will not be in utero for the full period for which weather conditions are included in the third trimester variables. However, we ultimately determined that it made sense to use a fixed exposure period in analysis – to avoid circularity in allowing the outcome to influence the length of the exposure period. We could either define trimester dates based on date of birth or date of conception, both of which would include data on exposure that does not occur during pregnancy for babies born before 39 weeks. The reason for choosing to measure from date of conception is that there is some research showing that exposures even pre-conception may affect birth outcomes, whereas it is not possible for exposures after birth to affect birth outcomes. So we decided that while defining exposure based on date of conception may introduce some noise by including irrelevant data, is less likely to lead to bias or misinterpretation than the alternative.

An alternative approach – used in much of the epidemiological research that focuses on the impact of heat exposure on early labour – is to use survival analysis. However, given that we are interested in exposure throughout pregnancy and not just the exposure that leads to early labour, we have not done this.

We have added the following sentence to the Data section to make this approach clear:

*“We link data based on date of conception, such that the first trimester is the first 12 weeks from the conception date, the second trimester is the following 14 weeks, and the third trimester is the following 13 weeks. Definitions for timing and length of trimesters vary across contexts, but these are the definitions commonly used in Australia. We analyse 39 weeks of gestation, as this is the average gestational length in our population.” [pg 16]*

**4.The use of fixed effect model**
By including the location-month-gender FE, the model is essentially comparing boys (girls) born in the same month and location. I would expect these fixed effects to absorb much variation that you would like to use for identification. Can you explain, e.g., using an example, what you are comparing conditional on these fixed effects?

Yes this is very true, that we would like to be able to use a lot of the variation for identification. But we use these interacted fixed effects to ensure that we’re absorbing as much of the variation as we can that’s not due to day-to-day, unexpected, fluctuations in the weather.

We have included the following text in the Analytical methods section of the manuscript to provide an example:

“*To illustrate what we are comparing with these community-month-sex, consider two boys born in Darwin in January of the same year. Both boys were conceived in the same location (Darwin) and the same month (April), so they share the same location-month-gender fixed effect, though they were conceived two weeks apart. They also share the same month-year fixed effects. However, their mothers experienced different heat exposure during pregnancy due to particularly warm weather occurring in January while one baby remains in utero but the other has already been born. The fixed effects allow us to compare outcome for these two boys, attributing any difference to the variation in heat exposure their mothers experienced, while holding constant all other factors that vary systematically by location, month of conception, and baby's sex.*

*This day-to-day variation in weather within the same location-month-gender group is the source of identification in our model. It represents truly exogenous variation—mothers cannot anticipate or control whether their pregnancy will coincide with an unseasonably hot days versus a cooler days within the same general season and location.*

*Our choice of interacted fixed effects follows prior studies (e.g. Cil and Kim 2022; Isen, Rossin-Slater, and Walker 2017). That said, Appendix C shows that our estimates are similar regardless of the specifics of how we define these fixed effects.” [pp 24-25]*

Also, can your data track the location of the mother over the pregnancy cycle? Or are you assuming the location of the mother does not change? It seems to be an important assumption to explicitly discuss for the fixed effect model to control for omitted variable bias, such as potential sorting on place of residence and timing of conception.

We only have data on the place the mother lived at the time of birth. We therefore assume that this is the place she spent her pregnancy, but yes this was an unstated assumption and we now have now clarified this in the manuscript. Further linkage of admin data might make it possible to back out mobility rates, but unfortunately this is not possible with the data we have.

We have added the following clarification to the Data section:

*“We link these records to NASA’s daily weather reanalysis data, based on the mother’s place of usual residence at the time of the birth. We do not have data on place of residence throughout pregnancy, therefore we assume that the place of residence at birth corresponds with the place the mother spent most of her time during pregnancy.” [pg 16]*

**5.Sample period.**
Page 15, lines 298-301, the paper states: the administrative data is extracted from January 1994 to 31 December 2014, but the regression sample is from March 2000 to September 2009. I did not understand the justification behind this sample period selection.

Apologies for the confusion – we have deleted mention of data from 1994 to 2014 as this is irrelevant to the analysis. The reason we choose this subperiod for analysis is for consistency within a larger project (for which output will be published separately).

Minor comments

1.Page 11, line 213: The font style of channel “Maternal behavioral” should be the same as the previous two channels.

Thank you.

2.Figure 4, 7, 8: I recommend the authors consider breaking the coefficient for each trimester into a separate subfigure. The current deployment of the coefficient is quite messy and hard to interpret.

We have revised these graphs to 3 parallel panels.

3.Page 23, line 446, equation (1): I believe the usage of subscript itj for preterm_{itj} might be inappropriate as it could confuse people into thinking the data is individual-level panel data. For each baby i, there is no variation at t and j. I suggest just using preterm_{i} or preterm_{i(tj)}.

Thank you, we have made this change.

4.Why does Figure 1 use the data from 2020-2023 while the main regression uses 2000 to 2009?

The main regression uses data on birth outcomes that have been made available by the NT Department of Health for research. Data from 2020-23 are not yet available. However, Figure 1 uses the most recent data that were available, which can be downloaded directly from NASA. The aim of figure 1 is to make the point that these variables are not strongly correlated in tropical setting, and we do not expect these relationships to change over times, so we thought it made sense to use the most recent data available.

5.In the first sentence of the abstract: “The impact of prenatal heat exposure on birth outcomes is well-established, but what is it about heat that affects prenatal development?” It is not clear to me why heat that affects prenatal development has to do with the main goal of the paper.

Apologies, we are missing a logical link there in the abstract – the basis is that birth outcomes are determined by the baby’s cumulative health and development in utero, and hence heat exposure will affect prenatal development, which may (but will not always) manifest in birth outcomes. We have reworded this so that prenatal health is not mentioned in the abstract.

The abstract now starts with:

*‘The impact of prenatal heat exposure on birth outcomes is well-established, but what is it about heat drives this relationship?’*

6.Adding citation if you would like to speak more to the literature on environmental economics methodology literature.
Ghanem, D., & Smith, A. (2021). What are the benefits of high-frequency data for fixed effects panel models?. Journal of the Association of Environmental and Resource Economists, 8(2), 199-234.

Thank you.

# Reviewer 2

The authors of this paper seek to answer the question of how heat exposure affects preterm birth by influencing fetal development through various pathways. While explaining their choice of heat metrics, the authors clearly establish that metrics appropriate for tropical climates may not be suitable elsewhere; for example, minimum temperature is not a reliable proxy for maximum temperature. This highlights the importance of carefully considering which aspect of heat exposure is being measured.

The conceptual framework, detailing how heat exposure may affect the fetus through heat stress, heat strain, and changes in maternal behavior, is clearly articulated. The paper also considers which specific components of heat, such as air temperature, humidity, or other environmental contributors, are most relevant for prenatal development.

The paper makes two main contributions. First, it quantifies five different ways of measuring heat exposures. Second, it evaluates which of these measurement choices matters most for preterm birth. The findings using the preferred heat exposure metric suggest that incorporating both daily maximum and minimum lead preterm birth, which adds to understanding that both moderate and extreme heat affect fetal development.

General Comments:
1. Good work

Thank you!

2. As the non-Aboriginal population tends to live in arid areas, which are more adapted to heat exposure, this may explain why they are not as strongly affected, as shown in Appendix E1, Column 1, and Appendix E2, Column 3.

Yes this is a good point – though it’s quite difficult to disentangle. It may be adaptation, it may be cultural practices and housing, or it could be just more noise (Aboriginal people live in communities scattered throughout the NT, while most non-Aboriginal people in the NT live in Darwin). It could also be birth outcomes are already substantially worse among Aboriginal babies, and hence the determinants are just more complex and less likely to be impacted by subtle exposures. We included this analysis because it is standard to split analysis by Aboriginal and non-Aboriginal populations in the NT, and this is expected by the government departments that provide the data we use. However, a paediatrician familiar with the context advised us not to speculate too much on this, given the many possibilities and complex determinants of health in Aboriginal communities. We have added the following text:

*“It may be that Aboriginal communities and cultural practices are better adapted to hot weather, leading to little impact of a marginal increase in heat exposure. It may also be the lack of statistically significant effects reflects the greater diversity among Aboriginal mothers in their exposures to heat due to the varied locations of Aboriginal communities – leading to estimates that are generally of similar magnitude to those for non-Aboriginal mothers, but less precisely estimated (Doyle 2023).” [pp24-25]*

3. Not sure if an example of animal studies will be good in this study!

Yes we understand some readers might be uncomfortable comparing human babies to animals, and might question the generalisability. We do mention animal studies (e.g. pg 11, line 206 and while they are not necessarily directly generalisable, they are very valuable in understanding how and when epigenetic effects might manifest. The reason is that there have been a range of experimental animal studies which have been able to manipulate the heat exposure that mothers experience during pregnancy, and collect outcome data consistently. They do have their shortcomings (they are often quite small studies), but are really valuable in helping to build evidence behind biological mechanisms. We have edited the text to clarify the reason for citing animal studies.

In this section we have fleshed this out a little to explain the value of the animal studies:

*“Both channels have been evidenced in experimental animal studies – where researchers have been able to control heat exposure and fluid intake in a way that is not possible in epidemiological research.” [pg 11]*

4. Can you add max temperature bins ranges as well in a subsection of Max and Min, line 355
Done, thank you.

Clarification Comments:
5. Lines 283–284 mention that the sample includes babies born between March 2000 and September 2009, while lines 298–300 refer to birth records from the year 2017. Although a limited sample from March 2000 to September 2009 is acknowledged, line 302 states that the data analysis is prepared for the period 2023–2024, which creates confusion about the actual time frame of the data used. I think it would be helpful to have clarification on the final dataset, specifically, how many observations were ultimately used for the heat exposure analysis, how many birth records were matched, and other related details.

Thank you – added in. Our analysis sample is 95% of births in the NT during our analysis period.

The Data section now reads:

*“Our analysis sample includes 95 percent of all babies who were born in the NT and conceived between March 2000 and September 2009, a sample of 34,258 children. There were 35,899 babies born during this period. Our sample excludes 1,020 births for which the mother’s place of residence could not be geocoded and therefore could not be matched to weather data, and 621 births for which some covariates were missing.” [pg 15]*

6. I wonder, on average, how many births are recorded per 50x55 km unit?

This varies a lot depending on the location, and note that the units of weather data are distinct from the location FE clusters used in analysis.

For the 50x55km units, the largest has 13,704 (this covers most of Darwin). The smallest has 9. The average is 206 births per unit.

For the location FE clusters, the largest is Darwin, it’s just over 17,000. The smallest is a community for which there are just 6 births. The average is 311 births per unit.

We have added these averages to the manuscript:

On units:

*This gives a total of just over 500 cells throughout the NT, 166 of which have births within our analysis period – an average of 203 births per cell.”[pg 16]*

On clusters:

*“There are 106 such clusters in our data, with an average of 311 births per cluster.” [pg 24]*

7. On a spatial scale, is there within-weather variation in a 50×55 km unit? I'm wondering how much of the variation occurs within that area

Yes it is likely that there will be variation within these units that we don’t observe. Not all people living within the area will experience the same weather conditions. However, such variation will be small, and these grid weather data are the most precise ones available in the NT. Aside from urban locations like Darwin, it is also likely that each unit will contain just one community and hence even though there is variation across the unit, the temperatures experienced by the people within that unit will be the same because they live close together.

8. Also, do you think there may be heterogeneity by income levels as well? That is, does individuals have access to ways to mitigate heat exposure?

Yes, there will almost certainly be heterogeneity by income levels. As you say, higher income households are more likely to mitigate heat exposure – e.g. through use of air conditioning. Ownership of air conditioning is actually very widespread in this context ([according to the Australian Bureau of Statistics](https://www.abs.gov.au/AUSSTATS/abs@.nsf/allprimarymainfeatures/A38DDA7F40718E43CA25750E00112A97?opendocument) only 4% of households didn’t have AC as at 2014). Though higher-income households are more likely to use it more intensively. The challenge is that we do not have any data on income – this is due to the institutional setup in Australia, with state/territory governments holding data on health (which is what we have available for this analysis), while the federal government holds tax and transfer data. These have not been linked together. We very much hope that both datasets will be linked together one day, as this will allow for much more detailed analysis – instead of producing estimates of average effects across the population, we would be able to identify characteristics of households that are at higher or lower risk of poorer health at birth due to heat exposure.

The conclusion now acknowledges this (addition in bold):

*“A range of unobserved factors are likely to influence these thresholds in any given context. For instance, medical advice, culture, work norms for pregnant women, and adoption of air conditioning and other adaptations will affect the size of this relationship and the relative contributions of heat stress versus heat strain-related conditions.* ***Many of these factors are likely to vary on important dimensions that we cannot measure – for instance, across socioeconomic status and income levels.”*** *[pg 44]*

9. Do you observe how the main results differ between the Darwin area and the non-Darwin areas?

Good suggestion, we hadn’t tried this but we did it for the preferred heat metric and actually this reveals something quite interesting – that the coefficients on the daily minimum temperature exposure covariates are statistically significant in the Darwin subsample, but not in the rest of the NT. Other than that, the estimate look similar to our main estimates. This supports the idea that daily minimum temperatures are particularly important to measure separately in a tropical climate.

However, the problem with this analysis is that we don’t have our location-based fixed effects, since everyone in the Darwin subsample is in the same location unit. This is why we have decided not to include this analysis in the manuscript or appendices. In terms of the births covered, it’s effectively very close to the arid-tropical breakdown we present already, though that breakdown has the benefit of incorporating multiple location units within the tropical subsample, allowing for estimation with fixed effects.

10. Could you show the minimum and maximum correlations across the entire study area, instead of only for Darwin, to better assess whether multicollinearity might be biasing the results?

Yes it is technically possible, though doing this for all locations within the study area would produce hundreds of graphs so would be difficult to make sense of the findings. We also hold our analysis data in a different structure to the graphs shown in the introduction so they would not look comparable. However, as a comparison, below is the correlation graph for Alice Springs (which is the main city within the southern, arid part of the NT). Here you can see that while the correlation between daily max and daily min is higher in Alice Springs than in Darwin, it’s still not as high as many temperate cities (correlation coefficient of 0.89 – close to Paris, but below the other examples in Fig 1).

We have added this to Appendix A.

11. I am curious, have you tried incorporating linear trends?

We hadn’t tried it but did in response to your comment – we added in a linear time trend for month-year of conception. The coefficient on the linear trend is not statistically significant, and the coefficients on the heat exposure metrics are exactly the same as in our main specification – so we haven’t added this to the manuscript.

1. This may be less likely going forward given that daily satellite and reanalysis data are available globally, though researchers using historical data or weather station data may still need to make compromises.
